# The Origins of Specificity in the Microcin-Processing Protease TldD/E

**DOI:** 10.1016/j.str.2017.08.006

**Published:** 2017-10-03

**Authors:** Dmitry Ghilarov, Marina Serebryakova, Clare E.M. Stevenson, Stephen J. Hearnshaw, Dmitry S. Volkov, Anthony Maxwell, David M. Lawson, Konstantin Severinov

**Affiliations:** 1 Centre for Data-Intensive Biomedicine and Biotechnology, Skolkovo Institute of Science and Technology, 143026 Moscow, Russia; 2 Institute of Gene Biology of the Russian Academy of Sciences, 119334 Moscow, Russia; 3 Lomonosov Moscow State University, Department of Chemistry, A.N. Belozersky Institute of Physico-Chemical Biology, 119992 Moscow, Russia; 4 Department of Biological Chemistry, John Innes Centre, Norwich Research Park, Norwich NR4 7UH, UK; 5 Bionano Institute, Peter the Great Saint Petersburg State Polytechnical University, Saint Petersburg 195251, Russia; 6 Waksman Institute for Microbiology, Rutgers, The State University of New Jersey, Piscataway, NJ 08854, USA; 7 Lomonosov Moscow State University, Department of Chemistry, Analytical Chemistry Division, 119991 Moscow, Russia

**Keywords:** microcin B17, DNA gyrase, metalloprotease, CcdAB, X-ray crystallography, toxin-antitoxin, ribosomally synthesized modified peptides, RiPP, peptidase

## Abstract

TldD and TldE proteins are involved in the biosynthesis of microcin B17 (MccB17), an *Escherichia coli* thiazole/oxazole-modified peptide toxin targeting DNA gyrase. Using a combination of biochemical and crystallographic methods we show that *E. coli* TldD and TldE interact to form a heterodimeric metalloprotease. TldD/E cleaves the N-terminal leader sequence from the modified MccB17 precursor peptide, to yield mature antibiotic, while it has no effect on the unmodified peptide. Both proteins are essential for the activity; however, only the TldD subunit forms a novel metal-containing active site within the hollow core of the heterodimer. Peptide substrates are bound in a sequence-independent manner through β sheet interactions with TldD and are likely cleaved via a thermolysin-type mechanism. We suggest that TldD/E acts as a “molecular pencil sharpener”: unfolded polypeptides are fed through a narrow channel into the active site and processively truncated through the cleavage of short peptides from the N-terminal end.

## Highlights

• *E. coli* proteins TldD and TldE form a heterodimeric metalloprotease
• Binding of peptides within the active-site cleft is not sequence-specific
• Specificity is controlled through the access of substrates via a narrow channel
• Peptides enter the channel N-terminus first and are processively digested

## Graphical Abstract

**Figure:**
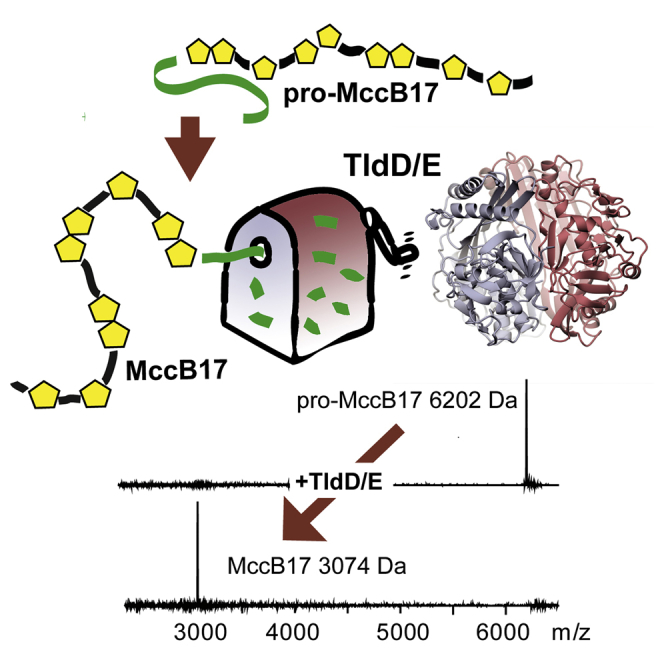

## Teaser

Ghilarov et al. crystallized the microcin-processing *E. coli* metalloprotease TldD/E in complex with various substrates, and used this structural information to explain the unusual specificity of the enzyme.

## Introduction

**Figure 1:**
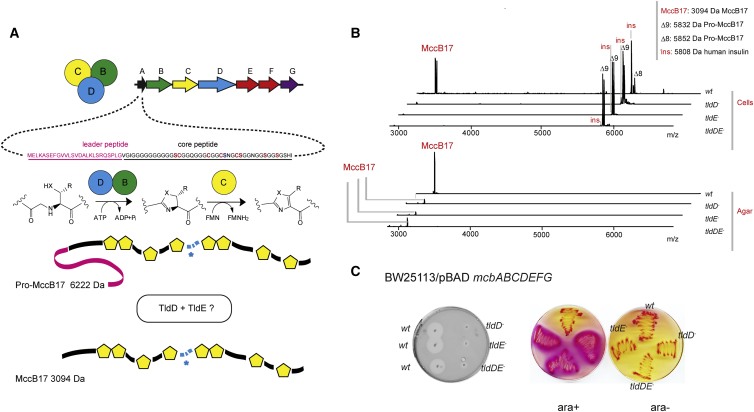
Tld Proteins Are Required for MccB17 Production *In Vivo* (A) An overview of MccB17 biosynthesis. The gene cluster *mcbABCDEFG* is annotated according to recommended nomenclature (Arnison et al., 2013). The leader peptide is shown in magenta and the core part in black, with the heterocyclizable residues highlighted in red. Ser52, which forms an ester bond, is highlighted in blue and marked with blue asterisk. (B) The accumulation of unprocessed heterocyclized MccB17 in *tld*^*-*^ cells and surrounding agar by whole-cell MALDI-MS imaging (top and bottom spectra, respectively). Equal quantities of cell material and agar were used and all samples were spiked with equal amounts of human insulin (*ins*) to serve as an internal reference. Full-size heterocyclized MccB17 precursor (*pro*-McbA Δ8-9, m/z = 5,832–5,852, reflecting different numbers of heterocycles formed) and the mature MccB17 (m/z = 3,094) are shown. (C) *Tld* deletion leads to the impaired export of active compound and accumulation of toxic heterocyclized MccB17 precursor inside the cells. Left: zones of bacterial growth inhibition on a soft agar inoculated with sensitive *E. coli* cells and used to overlay colonies of MccB17 producing *wt* and *tld*^*-*^*E. coli* strains (BW25113 pBAD-*mcbABCDEFG*). Right: SOS response visualized in *wt* and *tld*^*-*^*sfiA::lacZ* reporter strains, transformed by pBAD-*mcbABCDEFG* and grown on a MacConkey agar plate under inducing (1 mM arabinose) and non-inducing conditions. For clarity, only one of two identically inoculated plates is labeled.

*Escherichia coli* microcin B17 (MccB17) is a peptide antibiotic belonging to the linear azole-modified peptide family of natural products. MccB17 poisons *E. coli* DNA gyrase, ultimately leading to the accumulation of double-stranded DNA breaks and cell death (Heddle et al., 2001). MccB17 production and immunity require the *mcbABCDEFG* gene cluster (Genilloud et al., 1989) (Figure 1A). The *mcbA* gene encodes the 69-amino-acid long MccB17 precursor peptide, consisting of the leader and core parts; the latter part is subjected to post-translational modification by the McbBCD synthetase complex. The 26-amino-acid N-terminal leader peptide (McbA 1–26) serves as a recognition element for the binding of the synthetase complex (Roy et al., 1998, Sinha Roy et al., 1999). In the course of modification, all cysteine and most serine residues of the core peptide are converted to thiazole and oxazole heterocycles, yielding pro-MccB17 (Li et al., 1996). Most MccB17 molecules produced by cells harboring the *mcbABCDEFG* cluster contain eight (four oxazole and four thiazole) heterocycles and an ester bond, connecting residues Ser52 and Asp53 (MccB17 Δ8) (Ghilarov et al., 2011). Under the conditions of high expression of *mcbBCD*, an over-modified form of MccB17 containing an oxazole cycle at Ser52 (MccB17 Δ9) is also detected (Ghilarov et al., 2011, Sinha Roy et al., 1999). The last step in MccB17 maturation is removal of the leader peptide yielding mature MccB17, which is exported from the producing cell by the products of *mcbE* and *mcbF* genes (Garrido et al., 1988).

A screen for mutants abolishing MccB17 production identified TldE (PmbA) as a protein required for the cleavage of the MccB17 leader peptide (Rodriguez-Sainz et al., 1990). Allali et al. (2002) have presented *in vivo* data showing that *tldD*, a paralog of *tldE,* is also involved in MccB17 maturation. *E. coli tldE* and *tldD* were independently identified in several screens for mutants resistant to CcdB (LetD), an F-plasmid encoded toxin of the *ccdAB* toxin/antitoxin system (Murayama et al., 1996). CcdB inhibits DNA gyrase, and its toxicity is prevented by the binding of the CcdA antitoxin (Bahassi et al., 1999, De Jonge et al., 2009, Maki et al., 1992, Miki et al., 1984). The CcdA41 polypeptide, consisting of 41 C-terminal amino acids of CcdA, retains the ability to interact with CcdB and also prevents its cytotoxic activity (Bernard and Couturier, 1991). Mutations in *tldD* or *tldE* make cells more resistant to CcdB by affecting the stability of CcdA41 and, possibly, of CcdA (Allali et al., 2002).

Both *tldD* and *tldE* genes are common in prokaryotes, with 60% of bacterial genomes and almost all archaeal genomes encoding homologs of these two genes (Allali et al., 2002). The function of these highly conserved genes remains elusive. Comparative genomic analysis shows that *tld* genes are frequently located near *lon*, a gene that encodes an ATP-dependent protease, which, in *E. coli*, is involved in CcdA and CcdA41 degradation (Van Melderen et al., 1994, Van Melderen et al., 1996).

To date, structural information has only been available for the TldE protein, with four representative structures from *Thermotoga maritima* (PDB: 1VL4; Rife et al., 2005), *Pseudomonas aeruginosa* (PDB: 3QTD), *Shigella flexneri* (PDB: 3TV9), and *Bacteroides thetaiotaomicron* (PDB: 1VPB). All these proteins form roughly spherical homodimers with a hollow core and none possesses any recognizable catalytic site or shows biological activity. Given the relatedness between the sequences of TldD and TldE proteins, they are expected to share essentially the same fold, although TldD is distinct in that it contains a conserved metalloprotease-like HExxxH sequence motif that may be involved in catalysis (Hu et al., 2012).

In this work, we show that *E. coli* TldD and TldE interact to form a catalytically active metalloprotease capable of degrading unfolded peptides, including modified pro-MccB17 and CcdA41. By contrast, TldD/E is unable to process unmodified microcin B17 precursor peptide. In *tld*^−^ cells, large amounts of modified MccB17 precursor accumulate, indicating that leader peptide cleavage is necessary for toxin export. We present crystal structures of TldD/E protease alone and in complex with peptide substrates and inhibitors. These reveal that the heterodimer forms a spherical shell around a central cavity, analogous to the TldE homodimers reported previously, and that the conserved HExxxH motif in TldD does indeed coordinate a metal ion via the two His residues, together with an additional conserved Cys residue located near the C terminus. The substrate peptides are bound in an active site cleft on the inner surface of the TldD subunit, in a sequence-independent fashion via β sheet interactions. Based on structural superpositions with thermolysin we propose a metal-dependent proteolytic mechanism for TldD/E. Substrate access is via a narrow pore through the protein shell that leads directly into the active site. We hypothesize that the TldD/E substrate specificity is conferred by the size of the pore opening which restricts access to unfolded polypeptides only.

## Results

### Both TldD and TldE Are Required for MccB17 Leader Peptide Cleavage In Vivo

Previous reports (Allali et al., 2002, Rodriguez-Sainz et al., 1990) provide only limited data on the fate of MccB17 precursor inside the *tld* mutant cells. To get a clearer picture, we analyzed the TldD/E-dependent processing of MccB17 *in vivo* by MALDI-MS imaging of colonies of *E. coli* BW25113 cells transformed by plasmid pBAD-*mcbABCDEFG* expressing *mcb* genes from an inducible *ara*BAD promoter. MALDI-TOF spectra of induced wild-type BW25113 and *ΔtldE*, and *ΔtldDE* mutant cells transformed with pBAD-*mcbABCDEFG* differed dramatically (Figure 1B). Wild-type cells accumulated mature MccB17 Δ8 (m/z = 3,094 Da [M + H]+) and small amounts of MccB17 precursor with eight heterocycles (*pro*-MccB17 Δ8, m/z = 5,852 Da [M + H]+). In all mutant cells, hyper-modified *pro*-MccB17 Δ9 (m/z = 5,832 Da [M + H]+) MccB17 precursor was present. When the agar around cell colonies was analyzed, a very intense signal of mature (m/z = 3,094 Da [M + H]+) MccB17 was detected for wild-type, but not *tld* mutants (Figure 1B, bottom). Consistently, a dramatic decrease in the size of growth inhibition zones around colonies of *tld* mutants (compared with the wild-type) was observed, as previously reported (Figure 1C, left) (Rodriguez-Sainz et al., 1990). When a *sfiA∷lacZ* reporter strain and its *tld*^−^ derivatives (Allali et al., 2002) were transformed with pBAD-*mcbABCDEFG* and induced with arabinose, *tld*^−^ strains underwent SOS response, caused by the intracellular accumulation of *pro*-MccB17 Δ9 (Figure 1C, right) (Allali et al., 2002).

### Purified TldD/E Cleaves Modified MccB17 Precursor and Other Substrates In Vitro

**Figure 2:**
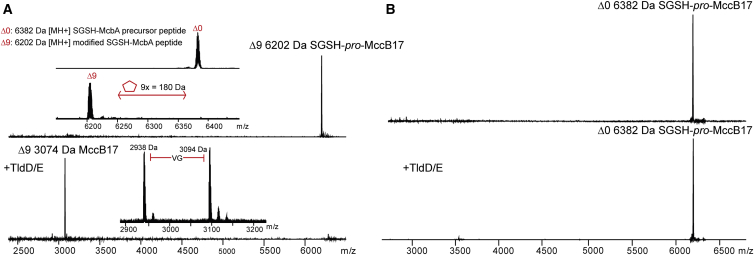
TldD/E Cleaves Modified Microcin B17 Precursor *In Vitro* (A) TldDE activity converts heterocyclized MccB17 precursor to the mature MccB17 molecule. Top: mass spectrum of nine-cycle containing heterocyclized MccB17 precursor (SGSH-*pro*-McbA Δ9); insert: mass spectrum showing introduction of nine heterocycles by MccB17 synthetase activity. Bottom: addition of TldD and TldE leads to the immediate disappearance of Δ9 mass peak and accumulation of 3,094 Da MccB17 product. In an insert: further cleavage of two amino acids (Val-Gly) from the N terminus of 3,094 Da MccB17. (B) Non-heterocyclized SGSH-*pro*-MccB17 precursor peptide (6,282 Da, top) is stable after overnight treatment with TldD/E (bottom).

N-Terminally 6xHis-tagged *E. coli* TldD and TldE proteins were separately purified by nickel affinity chromatography (see the STAR Methods). The expressed protein products had expected sizes on SDS gels (50.5 kDa for His-TldE and 53.5 kDa for His-TldD, respectively; see Figure S1A). Proteins were tested for their ability to cleave the likely substrate, SGSH-*pro*-MccB17 Δ9 (m/z = 6,202 [M + H]+, average mass). The substrate was obtained *in vitro* by overnight treatment of the recombinant SGSH-McbA peptide with *in vitro* reconstituted recombinant MccB17 synthetase (Figure 2A, top, insert) (see the STAR Methods for more details). A 2-hr incubation with either TldD or TldE alone did not alter the substrate. In contrast, the addition of both proteins together led to rapid disappearance of the 6,202 Da mass peak and accumulation of a 3,074 Da [M + H]+ product, which corresponds to mature MccB17 Δ9 produced by cleavage of the precursor between residues 26 and 27 (Figure 2A). At longer incubation times, a 2,918 Da [M + H]+ peak was also observed, corresponding to the MccB17 Δ9 variant lacking the first two residues from the N terminus (Val-Gly) (Figure 2A, bottom, insert). The purified TldD/E cleavage substrate SGSH-*pro*-MccB17 Δ9 is biologically inactive, presumably because it cannot be transported inside the susceptible cells, while TldD/E cleavage of SGSH-*pro*-MccB17 Δ9 produced active toxin (data not shown).

A mass peak corresponding to the leader peptide (MccB17 1–26) was absent from mass spectra of TldD/E reactions, probably due to extensive proteolysis. Indeed, purified MccB17 1–26 peptide (3,143 Da [M + H]+) was rapidly degraded by TldD/E (not shown). In contrast, when unmodified McbA precursor peptide was used, no cleavage was observed, either at the leader-core peptide junction site or within the leader peptide (Figure 2B). We conclude that TldD and TldE together act as a peptidase that processes the modified, but not the unmodified, McbA precursor. The negative result with unmodified precursor suggests that introduction of heterocycles changes the structure of McbA, unmasking cleavage sites in the leader and at the leader-core junction.

We also checked the ability of TldD/E to cleave its two other likely substrates, CcdA and CcdA41. Full-length SGS-CcdA was stable in the presence of TldD/E *in vitro* (Figure S2A), but SGS-CcdA41 was readily degraded to small peptides (Figure S2B). CcdA41 is an intrinsically disordered C-terminal tail of CcdA, and previous studies have shown that it is more susceptible to cleavage by Lon protease compared with the full-length protein, presumably due to the lack of interactions with N-terminal folded domain (Burger et al., 2017, Van Melderen et al., 1996). Hence our results support earlier western blot data showing stabilization of Ccd41 in *tld* mutants (Allali et al., 2002).

To find additional substrates, we tested TldD/E against a panel of synthetic peptides that are commonly used as MALDI calibration standards. The results are represented in Table S1 and Figure S1B. Most peptides were rapidly degraded; however, some remained intact even after overnight incubation with TldD/E. As with pro-MccB17, protease activity was manifested only in the presence of both TldD and TldE proteins. Analysis of proteolytic fragments gave no clear evidence for preferred sites of cleavage by TldD/E. The ability of TldD/E to cleave large proteins was tested using casein and native, reduced, and alkylated BSA, but no proteolytic activity was observed.

### TldD and TldE Form a Stable Complex

Having observed that *E. coli* TldD and TldE exhibit proteolytic activity *in vitro* only when present together, we analyzed the ability of these proteins to form a complex. During non-denaturing PAGE, TldD and TldE each migrate as sharp bands with distinct mobilities (Figure S3A). When the two proteins are combined together, a new lower mobility band appears. The analysis of the protein content of the native gel bands by SDS-PAGE (Figure S3A) showed that the shifted band consists of an apparently equimolar mixture of TldD and TldE, suggesting that the two proteins form a complex. Indeed, TldE was co-purified with TldD during affinity chromatography of extracts of cells co-overproducing His-tagged TldD and untagged TldE (Figure S3B); this purified complex produced a single peak during gel-filtration chromatography (Figure S3C). We used sedimentation equilibrium analytical ultracentrifugation (AUC) to measure apparent molecular weights of TldD, TldE, or of co-purified complex. In each case, only sedimenting species corresponding to protein dimers were detected (observed molecular weights: TldD, 115 kDa; TldE, 134 kDa; and TldD/E, 124 kDa). We therefore conclude that TldD and TldE can form both homo- and heterodimers.

### The Crystal Structure of TldD/E

**Figure 3:**
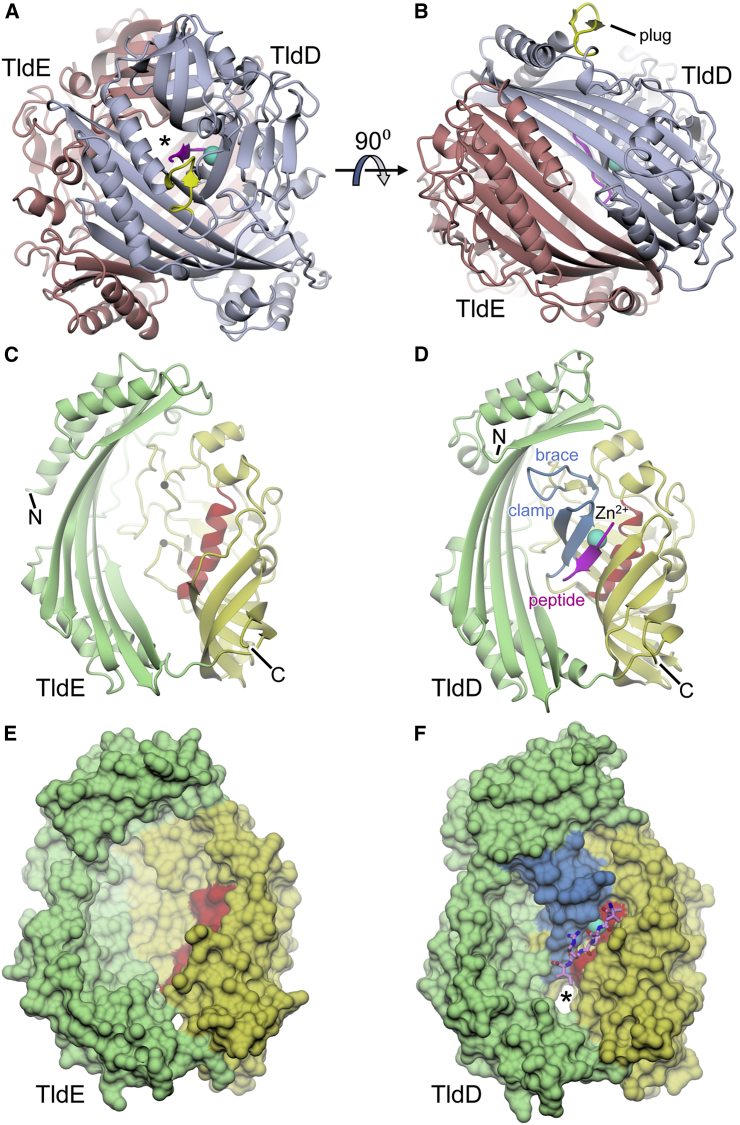
Overall Structure of the TldD/E Heterodimer (A and B) Orthogonal views of the TldD/E heterodimer shown in cartoon representation with TldD in slate blue and TldE in maroon; the non-crystallographic 2-fold axis is vertical in (A) and perpendicular to the plane of the image in (B). The catalytic zinc associated with TldD is represented by the cyan sphere and a bound peptide by the β strand in magenta. The putative substrate entrance channel is marked by an asterisk in (A); access through this channel may be mediated by the insert marked in yellow, which we describe as the “plug.” (C and D) Corresponding views of TldE and TldD subunits alone, as seen from the interior of the heterodimer, highlighting their similarities and differences. For each, the N-terminal domain is shown in green and the C-terminal domain in yellow. Within the latter, the central helical region (which includes the HExxxH motif in TldD) is highlighted in red. The black spheres in TldE indicate the approximate positions at which insertions arise to give the clamp and brace motifs shown in TldD; again the zinc is represented by the cyan sphere and a bound peptide by the magenta β strand. (E and F) Equivalent views to (C and D), but with the protein shown as a molecular surface and the bound peptide in stick representation with magenta carbon atoms; again the putative substrate entrance channel is marked by an asterisk.

The structure of the purified TldD/E protein complex was solved from a 1.9-Å resolution dataset (WT-PO4, Tables 1 and 2) using molecular replacement with templates based on structures that were 23% and 99% identical in sequence to TldD and TldE, respectively, to give two copies of the TldD/E heterodimer in the asymmetric unit. These are roughly spherical, resembling the previously crystallized TldE (PmbA) homodimers, with an average outer diameter of approximately 65 Å and a hollow core (Figures 3A and 3B). The sequence of *E. coli* TldE differs by only two amino acids from that of *Shigella flexneri* PmbA, and thus the *E. coli* TldE monomer superposes well upon a monomer from the *S. flexneri* PmbA homodimer (PDB: 3TV9; root-mean-square deviation [RMSD] = 0.84 Å). As described for *Thermotoga maritima* PmbA (PDB: 1VL4; Rife et al., 2005), the subunit is divided into two domains, each accounting for roughly half of the primary sequence. The N-terminal domain is pseudo 2-fold symmetric, being comprised of a very long and curved six-stranded anti-parallel β sheet with a pair of α helices at each end lying against the convex outer surface of the sheet. The C-terminal domain is made up largely of β strands, which are arranged into a five-stranded anti-parallel β barrel and a six-stranded, mainly anti-parallel, β sheet, which together form a funnel-shaped structure that wraps around a central α helix, although one face of the latter is exposed to the inner cavity of the heterodimer (Figure 3C). Despite only 18% identity between *E. coli* TldD and TldE, they share essentially the same fold with an overall RMSD of 2.53 Å (for 392 aligned residues) (Figure 3D). However, there are two notable insertions in the C-terminal domain of TldD giving rise to additional structural elements (see Figures 3, S4, and S5). One of these, which we term the “clamp” (residues 450–463), lengthens an outer strand of the six-stranded β sheet observed in TldE and adds an additional strand, giving a β hairpin that extends the sheet toward the lip of the β barrel, to leave only a narrow gap between the two motifs. The clamp is partly stabilized by the second insertion, which we call the “brace” (residues 351–361), that provides support against the inner surface of the N-terminal domain. Together, the clamp and the brace extend the “β funnel” to effectively enclose the central helix, with the exception of the narrow cleft that remains between the edge of the β sheet and the lip of the β barrel. Remarkably, apart from the four deposited TldE (PmbA) structures, there are no other proteins within the PDB that share any recognizable structural similarity to *E. coli* TldD or TldE.

**Table 1:** X-Ray Data Collection and Processing Statistics for TldD/E

Dataset^a^	WT-PO4	WT-DRVY	WT-HPF	WT-Act	E263A-hex	H262A-pent
Beamline	I04-1	I03	I03	I02	I04-1	I04-1
Wavelength (Å)	0.9200	0.9795	0.9763	0.9795	0.9282	0.9282
Detector	Pilatus 2M	Pilatus 6M	Pilatus 6M	Pilatus 6M	Pilatus 6M	Pilatus 6M
Resolution range (Å)^b^	75.29–1.90 (1.95–1.90)	74.77–1.25 (1.28–1.25)	86.76–1.40 (1.44–1.40)	50.87–1.50 (1.54–1.50)	65.29–1.35 (1.39–1.35)	65.16–1.42 (1.46–1.42)
Space group	P2_1_	P2_1_	P2_1_	P2_1_	P2_1_	P2_1_
a, b, c (Å)	64.77, 173.99, 83.52	64.63, 173.53, 82.66	64.63, 173.53, 83.06	64.57, 172.88, 82.63	65.29, 175.44, 84.33	65.16, 174.48, 83.65
α, β, γ (°)	90.00, 90.00, 90.00	90.00, 90.03, 90.00	90.00, 90.00, 90.00	90.00, 90.02, 90.00	90.00, 90.01, 90.00	90.00, 90.04, 90.00
Total observations^b^	569,651 (21,752)	3,363,286 (229,260)	2,398,303 (173,512)	1,948,868 (141,491)	2,787,364 (198,716)	2,365,696 (164,352)
Unique reflections^b^	136,171 (7,370)	500,650 (36,869)	349,069 (25,341)	287,508 (21,061)	403,735 (28,973)	346,357 (25,317)
Multiplicity^b^	4.2 (3.0)	6.7 (6.2)	6.9 (6.8)	6.8 (6.7)	6.9 (6.9)	6.8 (6.5)
Mean *I*/σ(*I*)^b^	12.3 (2.0)	8.5 (1.0)	9.6 (1.7)	10.1 (1.4)	17.0 (1.2)	11.9 (1.2)
Completeness (%)^b^	94.1 (69.1)	99.9 (99.7)	97.7 (95.6)	99.8 (99.2)	97.7 (94.6)	99.0 (98.0)
*R*_merge_^b^^,^^c^	0.087 (0.551)	0.134 (1.884)	0.115 (1.125)	0.133 (1.344)	0.067 (1.623)	0.084 (1.543)
*R*_meas_^b^^,^^d^	0.100 (0.683)	0.145 (2.057)	0.124 (1.217)	0.144 (1.457)	0.073 (1.755)	0.091 (1.677)
*CC*_½_^b^^,^^e^	0.997 (0.565)	0.998 (0.296)	0.997 (0.470)	0.997 (0.453)	0.999 (0.429)	0.998 (0.440)
Wilson *B* value (Å^2^)	18.2	9.1	12.1	10.1	13.6	15.3

a We have used a two-part dataset naming convention comprising the TldD variant type (WT, wild-type), plus a shorthand for the ligand bound at the active site, where PO4, phosphate; DRVY and HPF, sequences of angiotensin fragments; Act, actinonin; hex and pent, adventitiously bound hexa- and pentapeptides. N.B.: details of the H262A-pent structure are found in Figure S7.

b Values for the outer-resolution shell are given in parentheses.

c *R*_merge_ = ∑_hkl_ ∑_i_ |I_i_(hkl) − 〈I(hkl)〉|/ ∑_hkl_ ∑_i_I_i_(hkl).

d *R*_meas_ = ∑_hkl_ [N/(N − 1)]^1/2^ × ∑_i_ |I_i_(hkl) − 〈I(hkl)〉|/ ∑_hkl_ ∑_i_I_i_(hkl), where I_i_(hkl) is the ith observation of reflection hkl, 〈I(hkl)〉 is the weighted average intensity for all observations i of reflection hkl and N is the number of observations of reflection hkl.

e *CC*_½_ is the correlation coefficient between symmetry-related intensities taken from random halves of the dataset.

**Table 2:** Refinement Statistics for TldD/E Structures

Dataset	WT-PO4	WT-DRVY	WT-HPF	WT-Act	E263A-hex	H262A-pent
Resolution range (Å)^a^	75.29–1.90 (1.95–1.90)	74.77–1.25 (1.28–1.25)	86.76–1.40 (1.44–1.40)	50.87–1.50 (1.54–1.50)	65.29–1.35 (1.39–1.35)	65.16–1.42 (1.46–1.42)
Reflections: working/free^b^	129,447/6,723	475,619/25,030	331,643/17,425	273,176/14,330	383,738/19,995	328,992/17,364
Final *R*_work_^a^^,^^c^	0.139 (0.194)	0.149 (0.239)	0.130 (0.179)	0.130 (0.171)	0.145 (0.231)	0.138 (0.234)
Final *R*_free_^a^^,^^c^	0.178 (0.248)	0.182 (0.292)	0.171 (0.292)	0.169 (0.269)	0.183 (0.300)	0.171 (0.293)
Estimated coordinate error (Å)^d^	0.026	0.009	0.012	0.014	0.011	0.012
RMSD						
Bond (Å)	0.010	0.011	0.009	0.009	0.010	0.010
Angle (°)	1.38	1.52	1.40	1.39	1.38	1.35
No. of protein residues per chain (residue ranges)^e^	A: 480 (2–481) B: 444 (7–450) C: 479 (3–481) D: 444 (7–450)	A: 480 (2–481) B: 443 (8–450) C: 480 (2–481) D: 442 (9–450)	A: 480 (2–481) B: 443 (8–450) C: 480 (2–481) D: 444 (7–450)	A: 480 (2–481) B: 445 (6–450) C: 479 (3–481) D: 445 (6–450)	A: 479 (3–481) B: 443 (8–450) C: 480 (2–481) D: 442 (9–450)	A: 476 (2–122; 127-481) B: 444 (7–450) C: 480 (2–481) D: 445 (6–450)
No. of heterogen residues: Zn/ligand/water/other^f^	2/2/1,797/12	2/8/2,060/18	2/6/2,093/18	2/2/2,124/11	2/12/2,086/18	2/10/1,973/24
Mean B factors: protein/Zn/ligand/water/other^f^/overall (Å^2^)	22/16/25/30/32/23	12/8/13/24/17/14	16/13/17/26/21/18	13/11/17/26/21/15	17/19/26/27/21/18	18/17/24/32/23/21
Ramachandran plot (%)						
Favored/ allowed/disallowed (%)^g^	97.9/2.1/0.0	98.0/2.0/0.0	98.5/1.5/0.0	98.0/2.0/0.0	98.1/1.8/0.1	98.1/1.7/0.2
PDB accession code	5NJ5	5NJ9	5NJA	5NJB	5NJC	5NJF

WT-DRVY peptide ligand was refined with occupancy of 0.7. H262A-pent peptide ligand was refined with occupancy of 0.5.

a Values for the outer-resolution shell are given in parentheses. RMSD, root-mean-square deviation.

b The dataset was split into “working” and “free” sets consisting of 95% and 5% of the data, respectively. The free set was not used for refinement.

c The R factors *R*_work_ and *R*_free_ are calculated as follows: R = ∑(| F_obs_ − F_calc_ |)/∑| F_obs_ |, where F_obs_ and F_calc_ are the observed and calculated structure factor amplitudes, respectively.

d Based on *R*_free_ as calculated by REFMAC5 (Murshudov et al., 1997).

e The asymmetric contains two copies of the TldD/E heterodimer where chains A and C are TldD subunits, and B and D are TldE subunits.

f “Other” includes ethylene glycol, MES buffer, and sodium ions.

g As calculated using MolProbity (Davis et al., 2007).

### TldD/E Is a Metalloprotease Requiring Fe or Zn as Co-factors

Multiple sequence alignments against non-redundant sets of diverged but homologous sequences of TldD and TldE reveal (Figure S6, see also Hu et al., 2012) a strictly conserved putative zinc binding motif (HExxxH) in the TldD sequence. An identical motif is found in dipeptidylpeptidase III (DPP) (Baral et al., 2008) and a similar motif (HExxH) is present in thermolysin and matrix metalloproteases (MMPs) (Cerda-Costa and Gomis-Ruth, 2014, Holden and Matthews, 1988). We have measured the metal content of purified His-tagged TldD and TldE by inductively coupled plasma atomic emission spectroscopy (ICP-AES). In this technique, diluted protein solutions are vaporized and injected in a stream of plasma to produce radiation signatures specific for different elements. While TldE contained no metal ions, recombinant TldD contained roughly equal amounts of iron and zinc; the total metal content was approximately one mole of metal per mole of TldD monomer. To determine which of the two metal ions was required for enzymatic activity, we have grown TldD-overproducing cells on M9 minimal medium supplemented with 50 μM of zinc or iron salts. TldD purified from these cells contained, correspondingly, >95% zinc or >80% iron as measured by the ICP-AES. When the activity of these enzymes was measured, no difference was observed. We have checked the ability of EDTA, O-phenanthroline (OP), and several readily available metalloprotease inhibitors to block TldD/E action, using angiotensin II (Asp-Arg-Val-Tyr-Ile-His-Pro-Phe) as a test substrate (Table S3). OP and natural product actinonin (deformylase inhibitor) efficiently prevented angiotensin II hydrolysis by TldD/E. Interestingly, EDTA was ineffective at preventing hydrolysis even at high (100 mM) concentration; as were phosphoramidon and bestatin. Actinonin was therefore used to produce a co-complex structure with TldD/E (see below). The observed difference in reactivity between two metal chelators, EDTA and OP, is not unusual and can be explained by fast formation of an inhibitory ternary OP-metal-protein complex (Kleemann et al., 1986, Massaoud et al., 2011, Mayaux et al., 1982, Vallee and Hoch, 1957). In contrast, EDTA, a bulky ligand, primarily acts as metal ion scavenger (by chelating free metal ions in solution and thus slowly shifting the equilibrium toward apoenzyme); therefore, an enzyme with a sufficiently low metal dissociation constant is not inactivated. Indeed, we have found that removal of metal from TldD occurs very slowly and requires several rounds of dialysis against 10 mM OP to be complete. The enzymatic activity of the resultant apoenzyme can then be reconstituted by adding metal.

**Figure 4:**
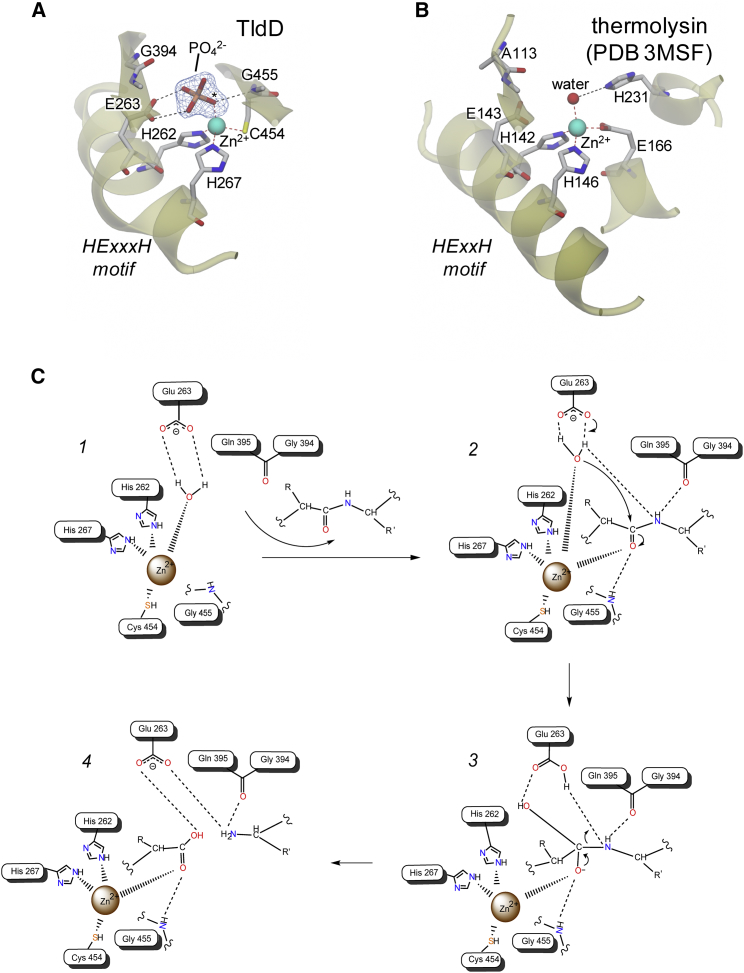
Comparison of the TldD Active Site with Thermolysin and Proposed Catalytic Mechanism (A) Close-up of the TldD active site showing key residues and a bound phosphate oxyanion superposed on a 1.9-Å resolution omit electron density map for the latter (contoured at ∼5σ). (B) Corresponding key residues taken from a representative thermolysin structure in the equivalent orientation to TldD shown in (A). Note the distortion of the helix in TldD resulting from the additional residue in the zinc binding motif. (C) Proposed promoted-water mechanism for TldD. (1) Situation prior to substrate binding with catalytic water bound to zinc and hydrogen bonded to the acid-base catalyst Glu263. The expected position of the water would overlap one of the phosphate oxygens shown in (A) (labeled with an asterisk). (2) The substrate is bound in the active site with the carbonyl oxygen of the scissile bond forming a fifth zinc ligand. Glu263 deprotonates the water molecule and the resultant hydroxide ion performs a nucleophilic attack on the carbonyl carbon. (3) The tetrahedral intermediate (mimicked by the phosphate complex) (A) breaks down as a result of protonation of the amide of the leaving group. (4) Situation post-reaction prior to dissociation of the products. For clarity, an interaction between Gly455 and the catalytic water is not shown in this scheme.

In the TldD/E structure, the TldD HExxxH motif, spanning residues 262–267, is located on the central helix that resides within the β funnel, and with the two histidine residues, His262 and His267, coordinating a metal ion, which we modeled as Zn (because Zn was added to the growth medium) (Figures 4A and S8); a third ligand is provided by Cys454 from the clamp domain. None of these residues are conserved in TldE, consistent with it being catalytically inactive. We have constructed His262 and His267 alanine substitution mutants, of which His267Ala was not folded correctly and His262Ala was catalytically inactive but, surprisingly, had wild-type Zn/Fe content as determined by ICP-AES. To rationalize this observation, we solved the structure of the mutant TldD^H262A^/E complex (Figure S7 and description), which revealed that the carboxylate group of the neighboring Glu263 replaces the missing imidazole moiety of residue 262 as the third TldD Zn ligand.

With the exception of the zinc binding motif, TldD and thermolysin share no obvious sequence or structural similarity (Matthews et al., 1972). However, it is possible to manually superpose the active site of TldD onto that of thermolysin such that the zinc ion and its liganding residues overlap closely; the only significant difference being that Cys454 of TldD serves as ligand in place of Glu166 in thermolysin (Figures 4A and 4B). In terms of the metal coordinating residues, TldD is even more akin to peptide deformylase, which also has a Cys as the third ligand (Chan et al., 1997). Despite their dissimilar folds, the zinc binding motifs of thermolysin and TldD, both lie in helical regions of their respective proteins, and these become aligned as a result of the above superposition. TldD accommodates the extra residue of its HExxxH motif (compared with HExxH of thermolysin) within a single helical turn between the two His residues, leading to a distortion of the helical geometry, similar to one observed in DPP; however, the observed distortion is even more pronounced in the case of TldD. By contrast, this distortion is not seen in the equivalent region of TldE. Importantly, our superposition also aligns Glu263 of TldD with Glu143 of thermolysin, which acts as an acid-base catalyst in the latter. In our first determined structure of TldD/E, a phosphate anion (derived from the crystallization solution, hence WT-PO4) serves as a fourth ligand to the zinc, which is also held by a bidentate interaction with Glu263. An oxygen atom of this bound phosphate corresponds to the position of the “catalytic” water molecule in thermolysin (Figures 4A and 4B). Taken together, these structural correlations allow us to propose a promoted-water mechanism (Xu and Guo, 2009) for TldD based on the wealth of data available for thermolysin and MMPs (Bertini et al., 2006, Cerda-Costa and Gomis-Ruth, 2014, Inouye et al., 2007) (Figure 4C). In the absence of adventitiously bound ligands, we expect the fourth zinc coordination site of TldD to be occupied by a water molecule. The carbonyl oxygen of an incoming substrate becomes a fifth zinc ligand, and the carbonyl carbon is then subject to nucleophilic attack by a hydroxide ion generated through Glu263-mediated deprotonation of the water molecule. The resultant negatively charged tetrahedral intermediate is stabilized through interactions with the zinc; this stage of the reaction is mimicked by the phosphate-bound TldD/E structure. The subsequent decomposition of the tetrahedral intermediate is initiated by Glu263-mediated protonation of the amide leaving group using the proton previously abstracted from the nucleophilic water (Figure 4C). Additional functional roles could be ascribed to the main-chain nitrogen of Gly455 (which overlaps with Nε2 of His231 in thermolysin) in binding the catalytic water and in stabilizing the tetrahedral intermediate, and to the carbonyl group of Gly394 (which overlaps with the carbonyl of Ala113 in thermolysin) in substrate binding and partial charge stabilization through hydrogen bonding to the amide nitrogen of the scissile bond (Figures 4A and 4B).

### The Substrate Binding Cleft of TldD/E Does Not Impose Specificity Constraints

**Figure 5:**
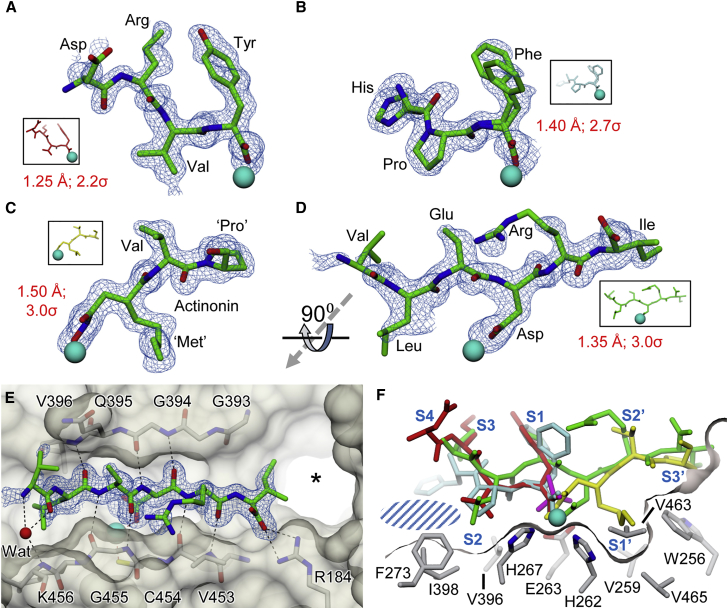
Ligand Complexes of TldD/E (A–D) Final coordinates of bound ligands shown in the same relative orientation (viewed from the side of the active site cleft) with associated omit electron density (resolutions and contour levels shown in red) and the active site zinc in cyan for (A) the N-terminal half of the cleaved angiotensin II peptide, (B) the C-terminal end of the angiotensin variant (the Phe side chain has been modeled in two alternative conformations), (C) the antibacterial agent actinonin (pseudo-residues labeled in quotes), and (D) the hexapeptide bound to the TldD-E263A mutant. (E) Detail of the hexapeptide (with omit electron density) in the context of the active site as viewed from above, showing the anti-parallel β sheet interactions that bind the peptide in the cleft that is highlighted by the semi-transparent molecular surface. The asterisk marks the putative substrate entrance channel. (F) Overlay of ligands shown in panels (A–D), colored and oriented as indicated in the small insets for these panels. In addition, the phosphate from the original 1.9-Å resolution structure is shown in magenta. The locations of the various subsites are labeled in blue; the blue hatched area indicates where there is scope for an expanded S2 subsite. The side chains of residues that delineate the subsites S2 and S1′ are shown; for clarity, Cys454 (the sulfur would eclipse the Zn in the current view) and Lys456 (which would be in the foreground providing one face of the P2 subsite) are not shown. The molecular surface is finely sliced to illustrate the contours of the substrate binding pocket without obscuring detail.

To explore the substrate binding site of the TldD/E heterodimer, wild-type or mutant (Glu263Ala) TldD/E was co-crystallized with a variety of peptides and peptide mimics, leading to four distinct ligand-bound complexes. Wild-type TldD/E crystals were grown in the presence of the peptide angiotensin II (Table S1), and customized angiotensin II variant where the central (Tyr-Ile) peptide bond is replaced with a non-cleavable methylene bond, to yield two co-crystal structures with clear density for peptides where the carboxyl group of the C-terminal residue acts as a ligand to the catalytic zinc ion. In the case of angiotensin II, the density was consistent with Asp-Arg-Val-Tyr (hence WT-DRVY; Tables 1 and 2; Figure 5A), indicative of cleavage at the central peptide bond of angiotensin. However, the methylene bond variant gave density for a His-Pro-Phe peptide, i.e., the C-terminal portion of the added compound (hence WT-HPF; Tables 1 and 2; Figure 5B); it was not clear whether the lack of clear density N-terminal to the His residue was due to a cleavage event, or simply that this region of the peptide was disordered. Using the standard nomenclature for protease active sites (Schechter and Berger, 1967), these two ligands spanned subsites S4-S1 and S3-S1, respectively, occupying roughly half of the active site cleft. By contrast, a wild-type crystal grown in the presence of the antibacterial agent actinonin (WT-Act; Tables 1 and 2) gave unambiguous density for the ligand in the other half of the active site cleft, chelating the zinc via its hydroxamate group, with the rest of the ligand mimicking a tripeptide spanning subsites S1′-S3′ (Figure 5C).

Attempts were made to trap uncleaved peptides in the active site of TldD through the use of a catalytically inactive Glu263Ala mutant. Surprisingly, the resultant density was essentially the same in every case, irrespective of the added ligand. We therefore focused on the highest resolution dataset, which had been collected to 1.45-Å resolution after co-crystallization with the engineered angiotensin variant described above. Clear density was present for the backbone of a hexapeptide spanning subsites S3-S3’ (hence E263A-hex; Tables 1 and 2) and, for four of the residues, probable identities could be assigned based on side-chain density to give an x-Leu-x-Asp-Arg-Ile sequence (Figures 5D and 5E). This bore no resemblance to any of the added ligands, but curiously gave a match with residues 49–54 of TldD itself (see Figure S3). A possible explanation for this could be that proteolytic fragments of the overexpressed TldD protein are comparatively abundant in the expression host, and this particular fragment binds with high affinity, principally due to the Asp residue in subsite S1′, the side chain of which does not occupy the pocket used by the n-pentyl chain of actinonin, but rather chelates the catalytic zinc.

It is especially apparent in this complex that the ligand binds in an extended conformation along the length of the active site cleft. Remarkably, hydrogen bonding is restricted to backbone atoms only, such that the peptide acts as an additional β strand that effectively fuses the β sheet and the β barrel motifs in the C-terminal domain of TldD through anti-parallel interactions with both supersecondary structural elements that extend from subsite S2 to S2′ inclusive (Figures 5D, 5F, and 3D). Furthermore, binding of the hexapeptide does not result in any significant conformational changes. When compared against the phosphate-bound structure, the RMSD after superposing TldD Cα atoms was only 0.33 Å (averaged across both copies of the subunit in the asymmetric unit). While it is not unusual for peptides to bind to proteins in extended conformations through β sheet interactions, being especially prevalent in the active sites of proteases (Malito et al., 2008, Tyndall et al., 2005), we are not aware of any other examples where the peptide unites two β-structural motifs by slotting into a pre-existing gap. The integrity of this cleft is maintained in part by the aforementioned brace that stabilizes the clamp against the inner surface of the N-terminal domain. Furthermore, a number of highly conserved residues play important roles. Among these are residues that help to tether the clamp, such as Cys454 and Lys456, both of which link β23 to the central helical region, the former via the active site zinc to His262 and His267 (in the HExxxH motif), and the latter via salt bridges to Glu270 and Asp272 that are also highly conserved (Figures S4 and S6). Indeed, alanine substitutions of Glu270 and Asp272 severely reduced expression of soluble protein (data not shown), consistent with an important structural role for these residues. Similarly, Arg362 in the brace provides further stabilization to the clamp via hydrogen bonds to backbone carbonyls in both β23 and β24.

As a consequence of this mode of binding, TldD has very relaxed substrate specificity. When all the ligand-bound structures are superposed, it is apparent that at most subsites, the peptide side chains either lie across the protein surface or are pointing away from it. Only the residues at subsites S2 and S1′ have their side chains directed toward the bottom of the active site cleft (Figure 5F). Subsite S2 is a shallow depression delineated by the side chains of Phe273, Val396, Ile398, Lys456, and His267, and would appear to be well suited to binding small or medium-sized aliphatic or neutral side chains. Indeed, our structural data indicate that it can accommodate all of Leu, Val, and Pro (Figure 5F); Leu and Val would also bind here when the two possible cleavages are made in the MccB17 precursor. However, from MALDI-MS data, it seems that this site is considerably more promiscuous, with Glu and Trp being accepted among others. It is possible that these bulkier side chains could be accommodated in a larger S2 subsite that extends beyond the lip of this shallow depression. Subsite S1′ is more enclosed and deeper, being bounded by the side chains of His262, Trp256, Val259, Val463, and Val465 (Figure 5F). Again this would appear to be well suited to binding small or medium-sized aliphatic or neutral side chains. In the actinonin structure, the n-pentyl chain of the ligand projects into the pocket, this being a mimic of a Met side chain. Moreover, for the two observed cleavage sites in the microcin precursor, Val and Ile would need to bind here. MALDI data indicate that Lys, Trp, and Glu are also accepted. Perhaps in this case, the subsite is able to remodel itself to accommodate these various side chains, e.g., a flipped Trp256 side chain would provide a significantly greater S1′ capacity.

### TldD/E Substrate Binding Specificity Is Governed by Compartmentalization of the Active Site

**Figure 6:**
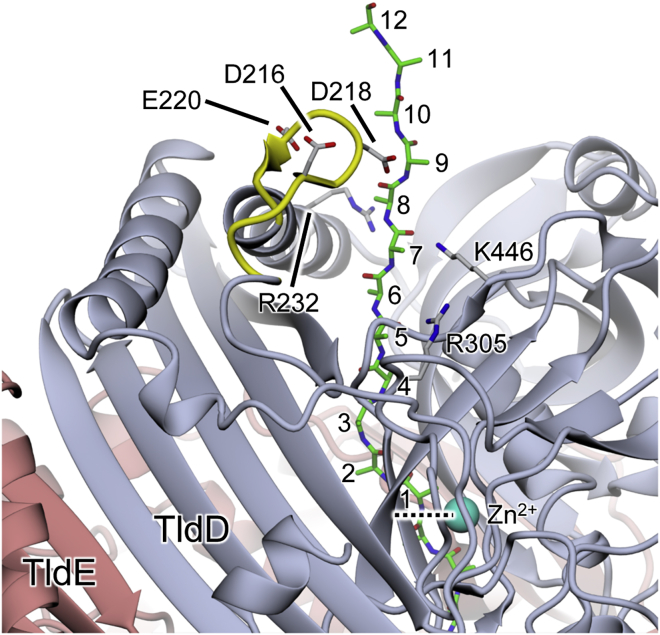
Substrate Access to the Active Site in TldD/E A modeled peptide (green carbons) stretching from the active site through the substrate access channel to the outside surface of the molecule suggests that an unfolded N-terminal region of at least 15 residues would be necessary in order observe proteolysis of an otherwise folded protein (assuming that the first six residues engage with subsites S3-S3′ and that a tripeptide is released). The site of proteolytic cleavage adjacent to the zinc (cyan sphere) is indicated by the dashed horizontal line. An insertion in the N-terminal domain of TldD relative to TldE gives rise to the short surface loop highlighted in yellow. The presence of three acidic residues in this loop and three basic residues arranged around the mouth of the channel (all labeled) suggests that under certain conditions the loop may “plug” the channel through the formation of salt bridges, thereby blocking access to the active site.

The active site cleft of TldD/E is open at both ends and, because of relaxed substrate preferences, any exposed protein terminus could be a potential substrate. Thus, TldD has the capacity to wreak proteolytic havoc within the cell, and it is presumably for this reason that the active site is sequestered within a protein shell, so that the availability of substrates is significantly restricted. We do not expect substrates to reach the active site through transient dissociation of the complex, since TldD/E heterodimer is stable during gel filtration and can withstand extensive high-salt washing (see the STAR Methods section). Instead, we propose that substrate peptides access the active site cleft by feeding through one of the handful of narrow channels perforating the outer shell of the heterodimer. The widest of these passes between the two domains of TldD and leads directly into the active site cleft, and is therefore the most likely access route. Given the orientation of the cleft relative to this channel, substrates entering the N terminus first would be correctly oriented to bind directly into the active site cleft. Thus, the unfolded N termini would be more likely to serve as substrates than C termini. It is difficult to envisage a situation where exposed surface loops could engage with the catalytic site at all. By modeling an extended peptide through the channel to the active site, we estimate that an unfolded N-terminal region of at least 15 residues would be necessary to achieve proteolysis of an otherwise intact globular protein (assuming that the first six residues engage with subsites S3-S3′ and that a tripeptide is released; Figure 6). Extensive degradation of the N-terminal McbA leader peptide after TldD/E treatment of modified McbA precursor peptide thus implies that the leader peptide is threaded through the channel and serially truncated until the heterocyclized region is sterically constrained by the entry channel.

While having an active site that is only accessible via a narrow channel would certainly restrict the range of potential substrates for TldD, there may be a further mechanism whereby substrate entry is controlled. In addition to the two inserts in the TldD sequence relative to TldE mentioned above, there is a third insertion that resides within the N-terminal domain. This forms a short loop in TldD that projects from the outer surface of the heterodimer adjacent to the putative substrate access channel. The loop contains three acidic residues (Asp216, Asp218, and Glu220), and there are three basic residues arranged around the mouth of the channel (Arg232, Arg305, and Lys446), prompting us to speculate that, under certain conditions, the loop may act as a “plug” to block the channel by the formation of up to three salt bridges with the channel mouth. Nevertheless, the main-chain temperature factors of the plug are not significantly elevated relative to the core of the molecule, suggesting that, at least in the crystalline state, this loop is not especially mobile.

## Discussion

With the exception of the four previously determined TldE structures and each other, *E. coli* TldD and TldE are not structurally similar to other known proteins. We show that they assemble to form a new class of heterodimeric metalloprotease, where the catalytic metal, which can be zinc or iron, is coordinated by residues of TldD only, via two His residues from a HExxxH motif and a remote Cys (residue 454). The proteolytic activity is only observed when both TldD and TldE are present. This is at odds with a recent observation that a *Sulfolobus solfataricus* TldD homodimer is catalytically active, and with the proposal that the equivalent of Cys454 is not a metal ligand, but instead forms a disulfide bridge in the TldD homodimer (Hu et al., 2012). From sequence comparisons it seems that in some cases Asp can substitute for Cys as the third metal ligand (e.g., in *Gemmatimonas* sp.), which would not be then capable to form any disulfide at all. Moreover, this substitution would give a more thermolysin-like active site, where the third ligand is Glu (Figure 4B).

We demonstrate that *E. coli* TldD/E cleaves off the N-terminal leader peptide of pro-MccB17, as well as a number of other peptide substrates. Crystal structures of TldD/E in complex with different peptide ligands demonstrate a unique binding mode in which the incoming substrate fits perfectly into a pre-existing gap between β strands, with hydrogen bonding restricted only to main-chain atoms. To counterbalance this relaxed specificity, the active center is enclosed within a spherical shell formed by the two partner proteins. Only completely unfolded peptides are allowed entry through a narrow channel leading directly to the active center cleft, an arrangement that would only support cleavage at the N terminus of the substrate. Since removal of the leader sequence is required before MccB17 can be exported, the inability of TldD/E to digest unmodified MccB17 precursor peptide ensures that only fully mature toxin is released. Intriguingly, the idea that MccB17 precursor has some degree of secondary structure in solution is indirectly supported by the observed N-C directionality of MccB17 biosynthesis (Kelleher et al., 1999, Roy et al., 1999), which can be explained by the progressive “unwinding” of the precursor peptide along with the incorporation of heterocycles. It is known that MccB17 biosynthesis (Kelleher et al., 1999) is distributive, meaning that McbBCD does not hold the substrate all the time. This should allow TldD/E to start processing as soon as MccB17 precursor with few heterocycles becomes available, exposing the leader sequence. This leads to the observed (Ghilarov et al., 2011, Sinha Roy et al., 1999) *in vivo* distribution of incompletely cyclized products exported from the cell.

**Figure 7:**
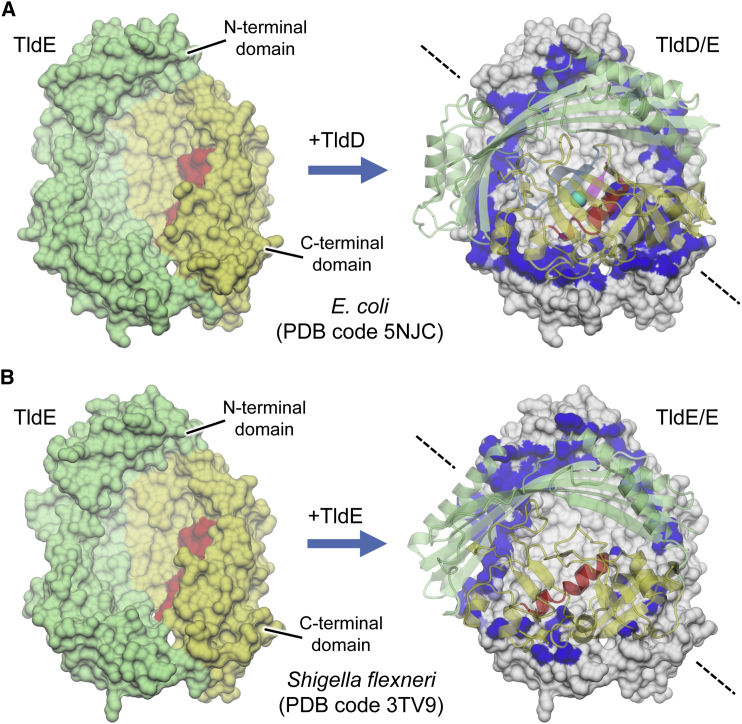
Dimer Interface in TldD/E Comparison of (A) *E. coli* TldD/E heterodimer and (B) *Shigella flexneri* TldE/E homodimer interfaces. In both cases the left hand panel shows a molecular surface for a TldE monomer viewed from the interior (oriented and colored as shown in Figures 3E and 3F). In the right-hand panels the same molecular surface is shown, but colored gray except for regions buried at the interface with the opposing subunit, where it is colored dark blue. Displayed in semi-transparent cartoon representation are the opposing subunits for each of the assemblies (colored as shown in Figures 3C and 3D) and, for TldD, the active site zinc (cyan sphere) and a bound peptide (magenta arrow) are also shown. The diagonal dashed lines indicate the approximate position of the 2-fold axes relating one subunit to the other. It is clear that the heterodimer (A) has a significantly more extensive interface than the homodimer (B), in particular where the β barrels of the C-terminal domains come together (bottom right patch; see also PISA analysis in Table S2).

We assume that one copy of TldD lost its catalytic center and evolved separately as the TldE protein. Lacking a catalytic center, TldE does not form a substrate access channel but instead provides extra space to accommodate the N termini of peptides, which potentially contributes to the processivity of the TldD/E heterodimer that functions as a “molecular pencil sharpener.” One can hypothesize that the two proteins co-evolved to offer higher stability of the outer shell and tighter regulation of otherwise promiscuous peptidase. Indeed, as both TldD and TldE (as judged by AUC) are able to form homodimers, preferential assembly of a heterodimer in the presence of both subunits must be a consequence of its higher stability. Indeed, analysis of the TldD/E heterodimer interface with the PISA server (Krissinel, 2015) gives a buried surface area of approximately 2,600 Å^2^, compared with 1,900 Å^2^ calculated for the *Shigella flexneri* TldE homodimer (Figure 7; Table S2). The TldD homodimer is not stable enough to be detected on a non-denaturing gel or to be purified as a dimer from bacterial cells. Such preferential association of heterologous subunits allows for a hypothetical activity control mechanism in which, given a steady-high level of expression of TldE, even transient expression of TldD will lead to formation of active proteolytic complex. We speculate that TldD is intrinsically susceptible to cellular proteases in its monomeric form and is rapidly degraded; this would minimize the potentially harmful effects of exposing the promiscuous active site to multiple possible substrates. Indeed, this might explain the origin of the hexapeptide we see bound to the Glu263Ala TldD mutant.

The widespread presence of *tld* genes suggests that they have an important function in bacterial physiology which might include, but not be limited to, a role in protein quality control and in the activation and degradation of different natural products (MccB17), peptide-derived co-factors (Pqq), or toxin-antitoxin modules (CcdA). We speculate that the unusual properties of this novel class of protease, which can be stably expressed in high yield, could be exploited for future applications in the protein engineering and synthetic biology fields.

## STAR★Methods

### Key Resources Table

**Table:** 

REAGENT or RESOURCE	SOURCE	IDENTIFIER
**Bacterial and Virus Strains**		

*E.coli* MG1655	DSMZ	DSM-18039
*E.coli* XL1-Blue	Agilent	200249
*E. coli* DH5a	NEB	C2987
*E. coli* BL21 (DE3) Gold	Agilent	230132
*E.coli* CSH600 sfiA::lacZ *E.coli* CSH600 sfiA::lacZ *tldD-* *E.coli* CSH600 sfiA::lacZ *tldE-* *E.coli* CSH600 sfi::lacZ *tldDE*	Prof. Van Melderen, Universite Libre de Bruxelles	(Allali et al., 2002).
*E. coli* BW25113	Dr. Kirill A. Datsenko, Purdue University	(Datsenko and Wanner, 2000)
*E. coli* BW25113 *tldD-* *E. coli* BW25113 *tldE-* *E. coli* BW25113 *tldDE-*	This work	

**Chemicals, Peptides, and Recombinant Proteins**		

Angiotensin II analogue (DRVY-CH2-IHPF)	Cambridge Research Biochemicals	
Angiotensin II	Sigma	Sigma #A9525
Actinonin	Sigma	Sigma #A6671; CAS: 13434-13-4
Bruker Peptide Calibration Standard II	Bruker	Bruker Part number #222570
1M MMT buffer pH 4 and pH 9	Molecular Dimensions	MD2-62
50% v/w PEG 1500	Molecular Dimensions	MD2-250-6

**Deposited Data**		

Phosphate bound TldD/E structure	This work	5NJ5
TldD/E in complex with DRVY angiotensin II fragment	This work	5NJ9
TldD/E in complex with a non-cleavable angiotensin II analogue	This work	5NJA
TldD/E in complex with actinonin	This work	5NJB
TldD/E E263A	This work	5NJC
TldD/E H262A	This work	5NJF

**Oligonucleotides**		

Oligonucleotides for TldD cloning in PET28: TldD NdeI f 5’-ATAAATCATAGATGAGTCTTAACCTGGTAAGT G-3’ TldD XhoI rev 5’-ATA AATCTCGACTTCGAGTACCGCCAAC AG-3’ Oligonucleotides for TldE cloning in PET28: TldE NdeI for 5’-ATAAATCATATGATGGCACTTGCAATG AAAGTAATC-3’ TldE XhoI rev 5’-ATAAATCTCGACTTACTGTCCGGCGAT TTTCATC-3’ Additional TldD oligonucleotides for pColA cloning: TldD BamHI f 5’-ATAAATGGATCCATGAGTCTTAACCTGGTA AGTG-3’ TldD Hind III rev 5’- TAAGCTTTCAGCTACCCGTACCACCTA-3’	This work	
Oligonucleotides for TldD mutagenesis: H262A for 5’ -CCGGGCGTGCTGTTGGCTGAAGCGGTTGGTCAC -3’ H262A rev 5’-GTG ACCAACCGCTTCAGCCAACAGCACGCCCGG-3’ E263A for 5’-GGCGTGCTGTTGCATGCTGCG GTTGGTCACGGT-3’ E263A rev 5’-ACCGTGACCAACCGCAGCATGCAACAGCACGCC-3’	This work	
CcdA cloning: CcdA BamHI f 5’-TAATATGGATCCATGAAGCAGCGTATTACA GTGA-3’ CcdA Not I rev 5’-TAATATGCGGCCGCTCACCAGTCCCTGTTCTCGT-3’ CcdA41 fragment cloning: CcdA41 BamHI f 5’-TAATAAGGATCCATGCAGAATGAAGCC CGTC-3’	This work	
TldD and TldE deletions TldD del f 5’-GGCAGCCGTAAAAAATCCTCTACTGCAGTAACT AACGAGTAGCAAAAACGGTGTAGGCTGGAGCTGCTTC-3’ TldD del r 5’- CGTTCGTGCACGTAGAAA GATTAATTATCCTTCTGA AAATAGTGA AATTAATGG GAATTAGCCATGGTCC -3’ TldE del f 5’-GCGCACTGAAAAACGGTTCTCTGTTAGACTTCAGAGAAACTCTCTACATTGTGTAGGCTGGAGCTGCTTC-3’ TldE del r 5’-GATTTTGTGTAATTTTTTAGTTTATAGCGCGGCAGGTCGCGCCAGTTTTTATGGGAATTAGCCATGGTCC-3’	This work	

**Recombinant DNA**		

Pet-28b (+)	Novagen	69865
pColA Duet-1	Novagen	71406
pKD4, pKD46, pCP20	Dr. Kirill A. Datsenko, Purdue University	(Datsenko and Wanner, 2000)
pBAD Ec-McB (pBAD *mcbABCDEFG)*	Dr. Mikhail Metelev, Skoltech	(Metelev et al., 2013)
pET28-MBP mcbA, B, C, D	Dr. Andrew Markley, UCSD	(Lee et al., 2008)

**Software and Algorithms**		

XDS	http://www.ccp4.ac.uk/	(Kabsch, 2010)
AIMLESS	http://www.ccp4.ac.uk/	(Evans and Murshudov, 2013)
XIA2	http://www.ccp4.ac.uk/	(Winter, 2010)
BUCCANEER	http://www.ccp4.ac.uk/	(Cowtan, 2006)
PHASER	http://www.ccp4.ac.uk/	(Mccoy et al., 2007)
CHAINSAW	http://www.ccp4.ac.uk/	(Stein, 2008)
REFMAC5	http://www.ccp4.ac.uk/	(Murshudov et al., 1997)
COOT	http://www.ccp4.ac.uk/	(Emsley and Cowtan, 2004)
PARROT	http://www.ccp4.ac.uk/	(Cowtan, 2010)
CCP4MG	http://www.ccp4.ac.uk/	(McNicholas et al., 2011)

**Other**		

LithoLoops 0.02 mm	Molecular Dimensions	MD7-130
SeedBead Kit PTFE	Hampton Research	HR2-320
96 well MRC crystallization plates	Molecular Dimensions	MD11-00-100
Amicon concentrators 30 kDa cutoff	Millipore	UFC903024
C18 Zip-Tips	Milllipore	ZTC18S096

### Contact for Reagent and Resource Sharing

Further information and requests for resources and reagents should be directed to and will be fulfilled by the Lead Contact, Ghilarov Dmitry (dmitry.ghilarov@uj.edu.pl)

### Experimental Model and Subject Details

*E. coli* DH5a (NEB) was used for all cloning purposes; BL21 (DE3) Gold (Agilent) was used for protein expression. CSH600 sfiA::lacZ reporter strain and its *tld*^*-*^ derivatives were described elsewhere (Allali et al., 2002) and are a gift of Dr. Van Melderen (Laboratoire de Génétique et Physiologie Bactérienne, Université Libre de Bruxelles, Gosselies, Belgium). BW25113 strain was a gift of Dr. Kirill A. Datsenko (Department of Biological Sciences, Purdue University, West Lafayette, USA).

### Method Details

#### Gene Disruptions

BW25113 Δ*tldD* and Δ*tldE* strains were produced by gene disruptions according to the published protocol (Datsenko and Wanner, 2000) using plasmids pKD4 and pKD46 (gift of Dr. Datsenko) and cleaned from the *kan* resistance cassettes by using the published FLP recombination protocol and corresponding plasmid pCP20 (Datsenko and Wanner, 2000) (gift of Dr. Datsenko). *tldDE* double deletion strain was obtained by the replacement of *tldE* gene in the cleaned *tldD* strain by the *kan* resistance cassette and its subsequent elimination.

#### Cloning and Purification of MBP-tagged Proteins

CcdA and CcdA41 sequences were amplified from the F-plasmid containing XL1-Blue *E. coli* strain (Agilent) and cloned to the pET28-MBP plasmid by using *Bam HI* and *Not I* restriction sites. A DNA fragment encoding for MccB17 leader peptide (McbA 1-26) was PCR amplified from *pBAD-mcbABCDEFG* plasmid and cloned into PET28-MBP by using *Bam HI* and *Not I* restriction sites. A full-length McbA peptide was cloned similarly. For purification of MBP-tagged proteins, 400 ml of BL21 (DE3) Gold, transformed with the relevant plasmid, were grown in 2YT medium to OD_600_ = 0.6, then induced with 0.5 mM IPTG and allowed to grow for 3 h at 37°C (for MBP-McbA, MBP-CcdA, MBP-CcdA41 and MBP-McbA 1-26) or overnight at 22°C (for MBP-McbB, C and D). Cells were harvested by centrifugation at 4000 g, resuspended in MBP lysis buffer (50 mM Tris·HCl pH 7.5, 500 mM NaCl, 2.5% (w/v) glycerol and 0.1% Triton X-100, 2 mg/ml lysozyme) supplemented with protease inhibitors (Roche) and lysed by sonication. Cleared filtered lysates were applied to 5 ml MBPTrap column (GE Healthcare) and washed by 20 column volumes (CV) of washing buffer (50 mM Tris·HCl 7.5, 400 mM NaCl, 2.5% glycerol, 2 mM DTT). Proteins were eluted from the column with elution buffer (50 mM Tris·HCl pH 7.5, 150 mM NaCl, 5% glycerol, 2 mM DTT, 10 mM maltose), fractions containing the highest concentrations of protein were aliquoted and frozen in liquid nitrogen.

#### Cloning, Mutagenesis and Purification of TldD and TldE Proteins

The TldD and TldE gene sequences were amplified from *E. coli* MG1655 genomic DNA by using Thermo Phusion DNA polymerase and cloned into pET28 b (Novagen) protein expression vectors by using *Nde I* and *Xho I* restriction sites. For the purification, 400 ml 2YT cultures were grown at 37 C to OD_600_ = 0.5 and induced with 0.5 mM IPTG for 4 h. Cells were subsequently harvested by centrifugation and resuspended in lysis buffer (20 mM Tris·HCl pH 8, 400 mM NaCl, 10 mM imidazole, 2 mg/ml lysozyme, 1 mM PMSF, 0.1 % Triton X-100) and lysed by sonication. Proteins were loaded on columns containing 0.5 ml of pre-equilibrated Ni-NTA resin (Qiagen). Resin was washed with 20 CV of lysis buffer containing 20 mM imidazole (20 mM Tris·HCl pH 8, 400mM NaCl, 20 mM imidazole) and proteins were eluted with 250 mM imidazole. Purified proteins were dialysed against storage buffer (20 mM Tris·HCl pH 8.0, 200 mM NaCl, 50% glycerol) and stored at -20°C. TldD active site mutants were constructed by overlapping extension PCR, cloned and produced as described above.

To obtain different combinations of tagged and untagged TldD and TldE proteins for the co-purification experiments, TldD and TldE genes were cloned into pColA-Duet (Novagen) co-expression plasmid. Cloning to MCS I by using *BamHI* and *HindIII* restriction sites led to the expression of tagged protein, whilst cloning into the second MCS by *NdeI* and *XhoI* led to the production of an untagged protein. For TldD/E pull-down trials, cells were grown, lysed and loaded on a column as described above and washed with ∼20 CV of wash buffer [20 mMTris-Cl pH 8, 5% glycerol, 0.1% Triton X-100. 1 M NaCl and 50 mM imidazole] before eluting with 250 mM imidazole..

#### *In Vitro* McbBCD Synthetase and TldD/E Reactions

Reactions with MBP-tagged McbA and McbB, C and D proteins were performed as described before (Lee et al., 2008). Briefly, MBP tags were removed by 2 h treatment with TEV protease at 30°C; this lead to the extra SGS or SGSH sequence on N-termini of recombinant peptides. The heterocyclized MccB17 precursor was obtained by overnight treatment of 10 μM SGSH-McbA with an equimolar mixture of BCD proteins (2 μM each) in the reaction buffer (50 mM Tris·HCl pH 7.5, 125 mM NaCl, 20 mM MgCl_2_, 2 mM ATP, 10 mM DTT). TldD/E was added at 0.3 μM. Reactions were incubated at 37°C for 120 mins or overnight. Aliquots were taken every 30 mins for MALDI analysis and desalted using Zip-Tips C18 (Millipore).

#### MALDI-MS and MS/MS Analysis

1-μl aliquots of desalted *in vitro* reaction mixture or ∼0.2 μl of cells growing on M9 minimal medium were diluted in 10 μl of 0.5% TFA. 1 μl of the diluted sample was mixed with 0.5 μl of 2,5-dihydroxybenzoic acid solution (20 mg/ml in 30% acetonitrile, 0.5% TFA) and left to dry on the stainless-steel target plate at room temperature. For the measurement of MccB17 in agar around producing colonies, a small piece of M9 agar was cut and extracted by three volumes of 0.5% TFA for 1 hour then a 1-μl aliquot was used for measurement. MALDI-TOF MS analysis was performed on UltrafleXtreme MALDI-TOF-TOF mass spectrometer (Bruker Daltonik, Germany) equipped with Nd laser. The MH+ molecular ions were measured in reflector mode; the accuracy of monoisotopic mass peak measurement was within 30 ppm. Spectra were acquired by averaging of a minimum 1000 laser shots from “sweet spots” of matrix crystals. Spectra of fragmentation were obtained in LIFT mode, the accuracy of daughter ions measurement was within 1 Da range. Mass-spectra were processed with the use of FlexAnalysis 3.2 software (Bruker Daltonik, Germany) and analyzed manually.

#### Metal Content Measurements (ICP-AES)

Purified proteins (0.1 – 0.5 mg/ml) were dialyzed three times against 10 mM Tris·HCl pH 8.0 in deionized (mQ) water in the presence of 0.5% Chelex-100 (Bio-Rad) to remove trace metal ions from the buffer. An axial ICP-AES 720-ES spectrometer (Agilent Technologies, USA) was used for measurements with a low flow axial quartz torch with 2.4 mm inner diameter injector tube (Glass Expansion, Australia), a double-pass glass cyclonic spray chamber (Agilent Technologies), a OneNeb nebulizer (Agilent Technologies, USA), and a Trident Internal Standard Kit (Glass Expansion). Samples were introduced manually to reduce washing volume, without preliminary digestion or dilution. A Sc solution (20 ppm) internal standard was added to increase the accuracy of measurements. Results were collected and processed by ICP Expert software 2.0.5 (Agilent Technologies). An ICP-AM-6 standard solution, 1000 ppm (High Purity Standards) was used for calibration in the range 10–200 ppb.

#### Non-denaturing Electrophoresis & AUC

Purified TldD and TldE (see above) at ∼2 mg/ml were loaded onto native Tris-Glycine acrylamide gels (0.75 mm, 7%) and run at 150 V. To assess complex formation, TldD and TldE proteins were mixed in sample buffer (pH 6.8) and incubated at 37 °C for 10 minutes. Gels were stained with InstantBlue (Expedion). The bands of the complex were excised from the gel and loaded to the wells of a SDS-containing denaturing gel (10%).

For AUC experiments proteins (TldD, TldE or TldDE) at 0.2 mg/ml were dialyzed into 50 mM Tris·HCl pH 7.5, 100 mM NaCl. Sedimentation equilibrium experiments were performed using a Beckman XLA-I analytical ultracentrifuge equipped with a Ti50 rotor and absorbance optics. Samples were centrifuged at 10000 rpm at 20°C and the absorbance was measured at 275 nm. Data were fitted to a single species model using Ultrascan (Demeler, 2005).

#### Crystallization of TldDE

The pColA TldD-His/E co-expression plasmid was used for the large-scale production of TldD-His/E complex. Proteins were expressed in minimal medium supplemented by Zn in order to have a >90% Zn occupancy. Briefly, BL21(DE3) Gold cells were transformed with the pColA-TldD/E expression plasmid and 10 ml of an overnight culture in LB was used to inoculate 2 L of M9 (supplemented with 0.5% glycerol as a carbon source, 1 μg/L thiamine, 50 μM ZnCl_2_ and 50 μg/mL kanamycin). Cells were grown at 37 °C with shaking to OD_600_ = 0.8, then IPTG was added to a concentration of 0.1 mM and cultures were moved to 20 °C for overnight expression. After 20 h, cells were harvested by centrifugation at 4000 g and resuspended in lysis buffer (20 mM Tris·HCl pH 8.0, 200 mM NaCl, 10 mM imidazole, 5% glycerol). Cells were lysed using a high-pressure cell disruptor (Constant systems Ltd) and the lysate cleared by centrifugation (28 000 g, 45 min) and filtration (0.45 μm). An AKTA Pure FPLC was used for the subsequent purification. Cleared lysate was loaded onto HiTrap Chelating HP 5 ml column charged with Ni^2+^ and washed by ∼20 CVs of lysis buffer containing 20 mM imidazole before elution with 250 mM imidazole; eluted protein was immediately loaded onto a Sephacryl S-200 HR 16/60 gel-filtration column operating in the final storage buffer (10 mM Tris·HCl pH 8, 50 mM NaCl). Peak fractions were pooled. Protein concentration was measured with a DirectDetect IR-spectrometer (Millipore).

Crystallization experiments were performed at a protein concentration of approximately 7 mg/ml and at a temperature of 20°C. Screening was conducted by sitting-drop vapour diffusion in MRC 96-well crystallization plates (Molecular Dimensions) with a mixture of 0.3 μl precipitant (from both commercial and in-house screens) and 0.3 μl protein solution, using either an OryxNano or an Oryx8 crystallisation robot (Douglas Instruments). Promising conditions were optimised with the latter robot using the same crystallisation format. Two conditions yielded crystals that were subsequently used for the data collections described herein. Condition 1 comprised 20 mM sodium potassium phosphate, 20% (w/v) PEG 3350, whilst condition 2 comprised 20% (w/v) PEG 1500 in 0.1 M MMT buffer (malate-MES-Tris; Molecular Dimensions) pH 8.0; crystals from both conditions were cryoprotected in precipitant supplemented with 20% (v/v) ethylene glycol. Once condition 2 had been established, the reproducibility of crystallisations under these conditions was improved by microseeding. A seed stock was prepared using crystals from several drops, which were broken up by repeated pipetting action, then pooled and transferred to an Eppendorf tube containing ∼300 μl of the precipitant. The sample was vortexed with a Seed Bead (Hampton Research) to break the crystals up further. Then crystallisations were set up using the Oryx8 robot with drops comprised of 0.4 μl precipitant, 0.15 μl protein and 0.05 μl of this seed stock. This was crucial for obtaining diffracting crystals of the TldD^H262A^/E and TldD^E263A^/E mutant protein complexes (using a wild-type seed stock).

All the ligand complexes were produced by co-crystallisation using condition 2. The ligands were prepared in 100% DMSO at concentrations of either 50 mM or 100 mM (for actinonin) and added to the protein to a final concentration of 2.5 mM. Thus, the final concentration of DMSO was either 2.5% (v/v) or 5% (v/v) depending on the ligand. Crystallisations were then set up using the seeding procedure described above. The resultant crystals were cryoprotected in precipitant supplemented with both ethylene glycol and the appropriate ligand.

Crystals were harvested and flash-cooled in liquid nitrogen using LithoLoops (Molecular Dimensions). The mounted crystals were stored in Unipuck cassettes (MiTeGen) prior to transport to the Diamond Light Source (Oxfordshire, UK), where they were transferred robotically to the goniostat on either beamline I02, I03 or I04-1 and maintained at -173 °C with a Cryojet cryocooler (Oxford Instruments). X-ray diffraction data were recorded using either a Pilatus 2M or 6M hybrid photon counting detector (Dectris), then integrated using XDS (Kabsch, 2010) and scaled and merged using AIMLESS (Evans and Murshudov, 2013) via the XIA2 expert system (Winter, 2010); the resultant data collection statistics are summarized in Table 1.

#### Structure Solution and Refinement

The TldD/E structure was solved by molecular replacement using a dataset collected to 1.9-Å resolution from a crystal grown in the phosphate buffer conditions. Initially, the data were processed in the orthorhombic space group P2_1_2_1_2_1_. However, it subsequently became apparent that the data were pseudo-merohedrally twinned (with operator h, -k, -l) and the true space group was in fact P2_1_ with β∼90°. As a result, all refinement was performed with REFMAC5 (Murshudov et al., 1997) using the “amplitude based twin refinement” setting and, in order to avoid bias resulting from the twinning, the R-free set of reflections was generated in the apparent orthorhombic space group before expanding to the true monoclinic space group; this R-free set was used throughout refinement for all datasets, which were isomorphous and similarly twinned. Molecular replacement templates were generated for TldE from *Shigella flexneri* PmbA (PDB code 3TV9; 99% sequence identity), and for TldD from *Thermotoga maritima* PmbA (PDB code 1VL4; 23% sequence identity) using CHAINSAW (Stein, 2008) where conserved side-chains were retained and all others were truncated to Cβ. PHASER (Mccoy et al., 2007) was able to locate two copies of the TldD/E heterodimer in the asymmetric unit (ASU), giving an approximate solvent content of 48%. After restrained refinement in REFMAC5, removal of badly fitting regions in COOT (Emsley and Cowtan, 2004) and further refinement, the R_work_/R_free_ values were 0.386/0.420 at 1.9-Å resolution. The model phases were then improved with PARROT (Cowtan, 2010) and used as input to completely rebuilding the model with BUCCANEER (Cowtan, 2006) which was able to fit 98.5% of the expected sequence for two copies each of TldD and TldE in the ASU, and gave much improved R_work_/R_free_ values of 0.260/0.312. The model was completed through several further iterations of model building in COOT and restrained refinement using isotropic thermal parameters with TLS group definitions obtained from the TLSMD server (Painter and Merritt, 2006). This model was used as the starting point for building and refinement of all the remaining structures. Each of these had been determined to resolutions of at least 1.5 Å and thus were refined in REFMAC5 using anisotropic thermal restraints. After rebuilding and refining the various structures, it became apparent that a point mutation had been introduced into TldD at position 401 such that the expected Gly had additional density consistent with the presence of a side-chain. The identity of the mutated residue was subsequently confirmed to be Asp by sequencing, which was then introduced into all the models. In all structures, there was residual density that could be attributed to ligands derived from crystallisation or cryoprotectant solutions, including phosphate, MES buffer, ethylene glycol and sodium ions. Several of these were modelled into the density, although those that did not refine satisfactorily were omitted from the final models. Final statistics for all models are reported in Table 2. Omit *mFobs-dFcalc* difference electron density maps were generated for bound ligands using phases from the final models without these ligands after the application of small random shifts to the atomic coordinates, re-setting temperature factors, and re-refining to convergence. All structural figures were generated using CCP4mg (McNicholas et al., 2011)

### Data and Software Availability

Coordinates and structure factors were deposited in the RCSB Protein Data Bank with accession codes 5NJ5 for the phosphate bound TldD/E, 5NJ9 for the TldD/E in complex with angiotensin DRVY fragment, 5NJA for the structure with a non-cleavable angiotensin analogue (angiotensin-HPF), 5NJB for the TldD/E in complex with actinonin, 5NJC and 5NJF for the mutant TldD^E263A^/E and TldD^H262A^/E complexes, respectively. All the used software is readily available.

## Author Contributions

D.G. and K.S. designed and planned the experiments with input from M.S., A.M., and D.L. M.S. collected and analyzed the mass spectrometry data. D.G. purified proteins and performed biochemistry experiments. D.V. collected ICP-AES data. D.G., S.H., and C.S. produced protein crystals. C.S. and D.L. collected the diffraction data, which were analyzed by D.L. D.G. and D.L. wrote the manuscript with input from A.M. and K.S.

## Supporting Citations

The following references appear in the Supplemental Information: Krissinel and Henrick, 2004, Robert and Gouet, 2014, Sievers et al., 2011, Waterhouse et al., 2009.

## Supplemental Information

Document S1. Figures S1–S8 and Tables S1–S3

Document S2. Article plus Supplemental Information
