# Supplementary material for: The Origins of Specificity in the Microcin-Processing Protease TldD/E
Source: Structure. 2017 Oct 3;25(10):1549–1561.e5. doi: 10.1016/j.str.2017.08.006 (PMC5810440; doi:10.1016/j.str.2017.08.006)
Supplement: Document S1. Figures S1–S8 and Tables S1–S3 [file mmc1.pdf]

**Structure, Volume *25***

## Supplemental Information

# The Origins of Specificity in the Microcin-Processing Protease TldD/E

**Dmitry Ghilarov, Marina Serebryakova, Clare E.M. Stevenson, Stephen J. Hearnshaw, Dmitry S. Volkov, Anthony Maxwell, David M. Lawson, and Konstantin Severinov**

**Table S1. Cleavage sites of TldD/E in tested peptides by the analysis of MALDI data. Related to Figure 2**

| **Peptide name** | **Observed cleavage sites (/)** |
|---|---|
| Angiotensin I | DRVY/IH/PFHL |
| Angiotensin II | DRVY/IHPF |
| Substance P | RPKDQQF/FG/LM |
| Bombesin | (pE)/QRLG/N/Q/W/A/VGH/LM-$NH_2$ |
| Somatostatin 14 | No cleavage |
| Bradykinin | No cleavage |
| Bradykinin 1-7 | No cleavage |
| ACTH18-39 | RPVKVYPNG/AEDESAEAFPLEF |
| ACTH1-17 | SYS/M/EHFRWG/KPV/G/K/KR |
| ACTH1-10 (human) | SY/SMEHFRWG |
| Somatostatin 25 | SAN/SNPA/MAPRERKAGCKNF/FWKTFT/SC |

**Table S2. Analysis of dimer interfaces with PISA. Related to Figure 7**

| Structure | Source | PDB code | Dimer[a] | Area[b] | ΔG[c] | P-value[d] | $N_{hb}$[e] | $N_{sb}$[f] | $N_{ds}$[g] |
|---|---|---|---|---|---|---|---|---|---|
| **TldD/E** | *E. coli* | 5NJ9 | AB | 2637.8 | -14.0 | 0.317 | 36 | 10 | 0 |
| | | | CD | 2615.0 | -15.6 | 0.223 | 34 | 7 | 0 |
| **TldE/E** | *S. flexneri* | 3TV9 | AA | 1910.9 | -14.7 | 0.188 | 27 | 8 | 0 |
| **TldE/E** | *T. maritima* | 1VL4 | AA | 1148.3 | -7.2 | 0.364 | 14 | 3 | 0 |
| **TldE/E** | *B.thetaiotaomicron* | 1VPB | AA | 1277.8 | -8.2 | 0.334 | 22 | 0 | 0 |
| **TldE/E** | *P.aeruginosa* | 3QTD | AD | 1460.2 | -8.2 | 0.268 | 32 | 7 | 0 |
| | | | BC | 1502.1 | -8.1 | 0.279 | 30 | 7 | 0 |

[a] Type of dimer: “AA” indicates that the asymmetric unit contains only one subunit (A chain). The biologically relevant dimer is created by applying two-fold crystallographic symmetry to this chain. For all other combinations, the asymmetric unit contains either one or two biologically relevant dimers, and the letters denote the chain names of the two subunits that form each dimer.
[b] Interface Area: half of buried surface area formed on interfacing monomeric units.
[c] Solvation energy gain upon interface formation (kcal/mol).
[d] Hydrophobic P-value of the interface: the lower P-value, the more specific, or statistically surprising, the interface.
[e] Number of hydrogen bonds formed between the interfacing monomers.
[f] Number of salt bridges formed between the interfacing monomers.
[g] Number of disulphide bonds formed between the interfacing monomers.
[b-g] Values calculated by the jsPISA server (http://www.ccp4.ac.uk/pisa) (Krissinel, 2015)

**Table S3. TldD/E inhibition data. Related to Figure 2 and Figure 5**

| **Inhibitors tested** | **Inhibition observed*** |
|---|---|
| EDTA (general chelator) | – |
| O-phenanthroline (general chelator) | + |
| Actinonin (deformylase inhibitor) | + |
| Benzhydroxamic acid (HDAC inhibitor) | – |
| Pro-Leu-Gly-NHOH (MMP1 inhibitor) | – |
| Z-Prol-Leu-Gly –NHOH (MMP1 inhibitor) | – |
| 4-aminobenzoyl-Gly-Pro-Leu-NHOH (MMP1 inhibitor) | + |
| Astatin (peptidase inhibitor) | – |
| Bestatin (peptidase inhibitor) | – |
| Phosphoramidon (potent inhibitor of thermolysin and other metallo-endopeptidases) | – |

***Positive*: a dominant peak of angiotensin peptide was present on MALDI after 10 min incubation with TldD/E at 37° C. *Negative* (uninhibited control): angiotensin peak was not visible. All inhibitors were tested at 1 mM concentration.

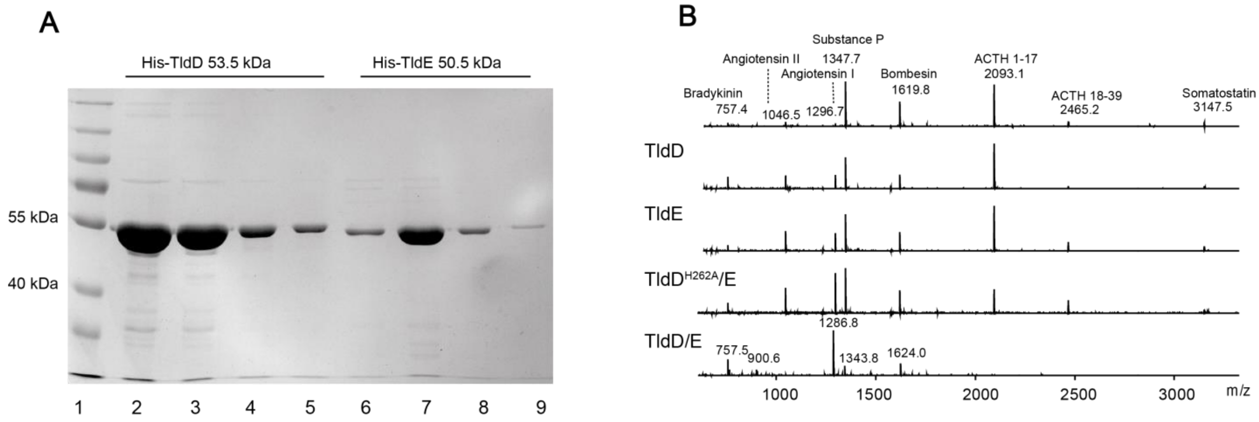


**Figure S1. Specificity of TldD/E activity. Related to Figure 2** (**A**) SDS-PAGE of fractions eluted from HiTrap Ni-NTA column during His-TldD and His-TldE purification. Lane 1 – molecular weight markers (PageRuler Prestained Protein Ladder). Lanes 2 to 5 – four consecutive fractions from His-TldD purification. Lanes 6 to 9 – four consecutive fractions from His-TldE purification. Molecular weights of His-TldD and His-TldE are 53.5 kDa and 50.5 kDa, respectively. (**B**) TldD/E activity evaluated by the ability to digest peptides used as MALDI calibration standards. From *top* to *bottom*: untreated peptide mixture with peptides and their measured masses indicated; mixtures treated with His-TldD, His-TldE, His-TldD$^{H262A}$active site mutant plus His-TldE protein (see also Figures S4, S6 and S7), and His-TldD plus His-TldE proteins. Masses of detected peptides are indicated. All masses are [M+H]+.

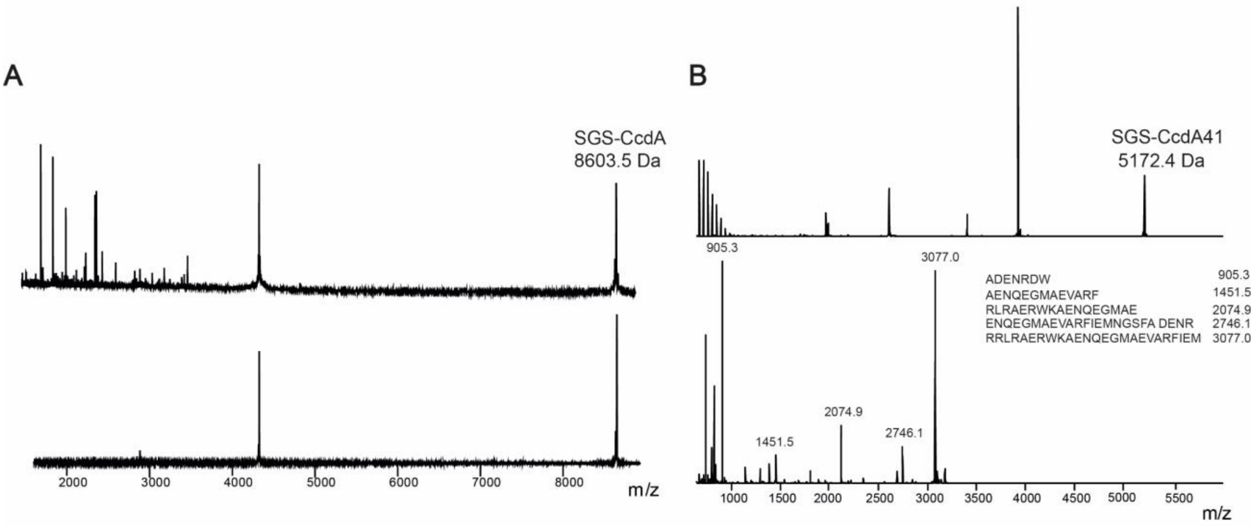


**Figure S2. CcdA degradation by TldD/E. Related to Figure 2** (**A**) Full-length SGS-CcdA, produced by TEV cleavage of MBP-CcdA fusion protein is stable after Tld treatment (compare top untreated and bottom treated spectra). Note the absence of some of the contaminant peptides in the bottom spectrum, which have probably been degraded by TldD/E. (**B**) SGS-CcdA41 peptide, produced in the same way, is rapidly degraded. The peptides shown were identified as fragments of SGS-CcdA41 by MS/MS analysis. Again, some of the contaminant peptides in the top spectrum have also been degraded by TldD/E.

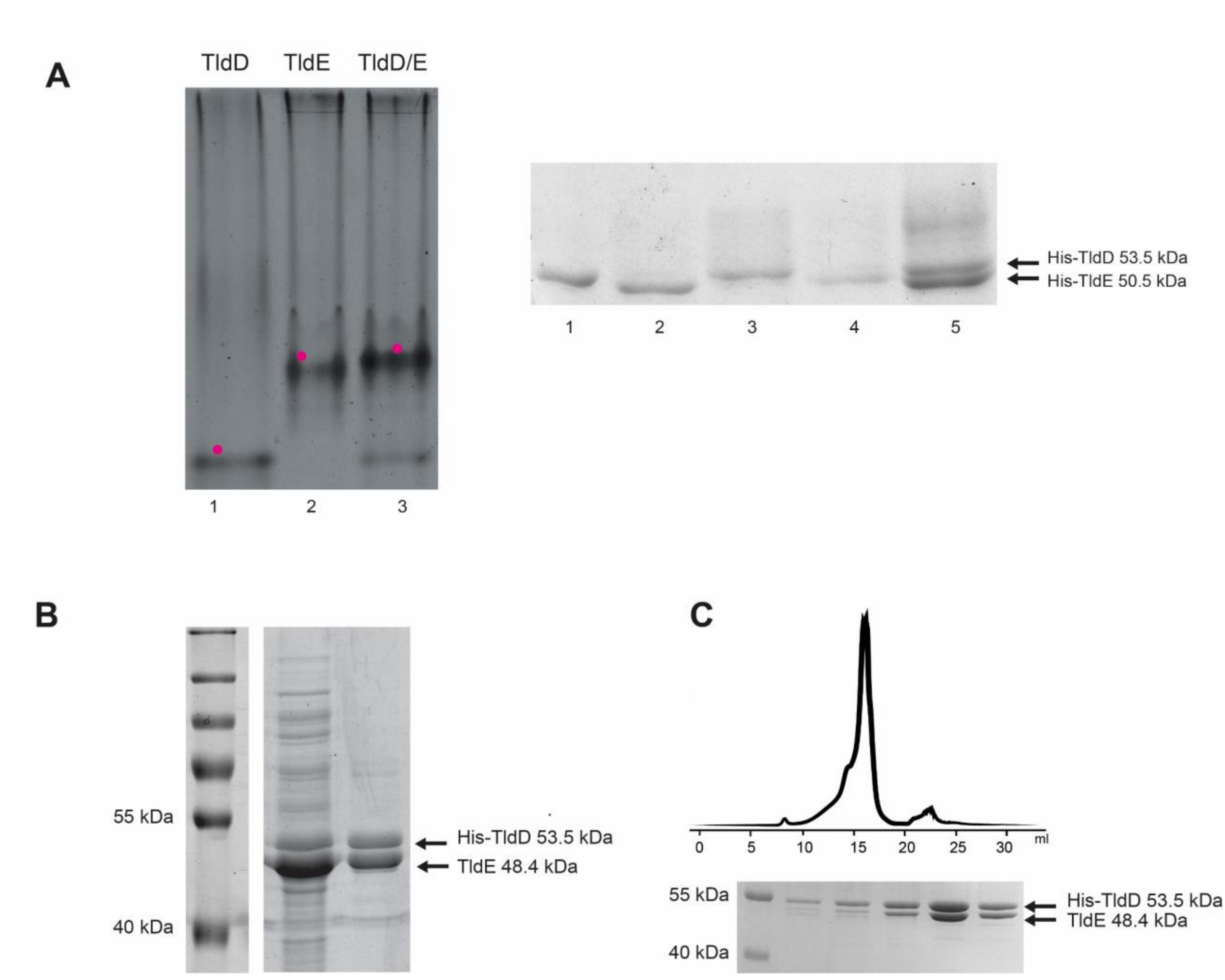


**Figure S3. TldD/E complex formation. Related to Figures 3 and 7** (**A,** *left*) A non-denaturing PAGE analysis of His-TldD (lane 1), His-TldE (lane 2) and His-TldD/His-TldE complex (lane 3). Specific bands (indicated by magenta dots) were cut from the native gel and loaded onto the denaturing gel (right) for analysis of their contents. (**A**, *right*) SDS-PAGE analysis of TldD/E complex. Lanes 1 and 2 – purified His-TldD and His-TldE, respectively. Lanes 3 and 4 – excised from (**A**, *left*) bands of TldD and TldE proteins, respectively. Lane 5 – a band of the TldD/E complex excised from lane 3 of the native gel. Molecular weights of His-TldD and His-TldE are indicated. (**B**) Affinity purification of His-TldD/E protein complex from bacterial cells. Protein molecular weight marker, clarified lysate and elution from $Ni^{2+}$ affinity column are shown (from left to right, respectively). Molecular weights of His-TldD and TldE are indicated. (**C**, *top*) gel-filtration trace (GE Superose 6 Increase 10/300) of affinity-purified His-TldD/TldE complex. (*bottom*) peak fractions loaded on a denaturing gel together with molecular weight markers (first lane); protein molecular weights are indicated.

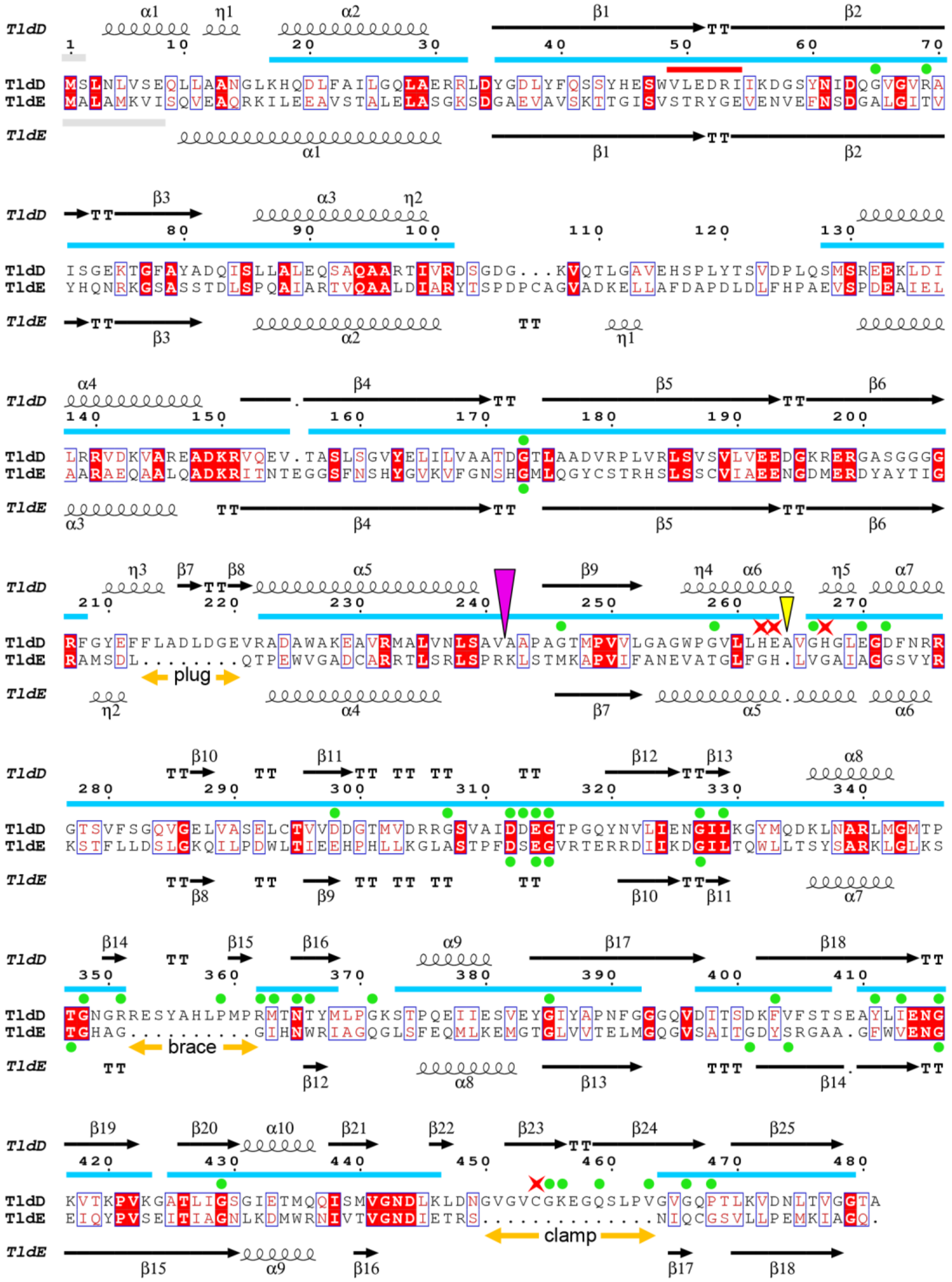


**Figure S4. Structure-based sequence alignment of TldD and TldE. Related to Figures 3, 4 and 7.** Shown are the full native sequences of TldD and TldE from *E. coli*, which were initially aligned using the PDBeFold server (http://www.ebi.ac.uk/msd-srv/ssm)(Krissinel and Henrick, 2004) based on subunits taken from the highest resolution TldD/E structure (PDB code 5NJ9). This was subsequently adjusted manually with reference to the superposed subunits, and then displayed using ESPript3.0 (http://espript.ibcp.fr/ESPript/ESPript) (Robert and Gouet, 2014). Strictly conserved residues are highlighted with red shaded boxes, and semi-conserved residues are coloured red. Analysis of these in relation to the structure revealed that virtually all of them could be assigned a clear structural and/or

functional role. Secondary structure elements for TldD are shown above the alignment, with those for TldE below, where α = α helix, β = β strand, η = $3_{10}$ helix, TT = β turn. The blue horizontal bars indicate the regions where the agreement between the two structures is good, the grey bars indicate the N-terminal regions that could not be modelled, and the red bar marks the possible origin of the hexapeptide fragment (residues 49 – 54) observed in the active site of the TldD-E263A mutant. The red crosses denote the zinc ligands and the catalytic glutamate, while green dots show highly conserved residues within the respective TldD and TldE families based on multiple sequence alignments of diverged and non-redundant sequence sets. Also labelled, with double-headed orange arrows, are the three main insertions in the TldD sequence relative to that of TldE. The magenta wedge shows the boundary between the N-terminal and C-terminal domains, and the yellow wedge indicates where a single residue insertion relative to TldE disrupts the helical region bearing the HExxxH motif in TldD (see Figure 4**A**).

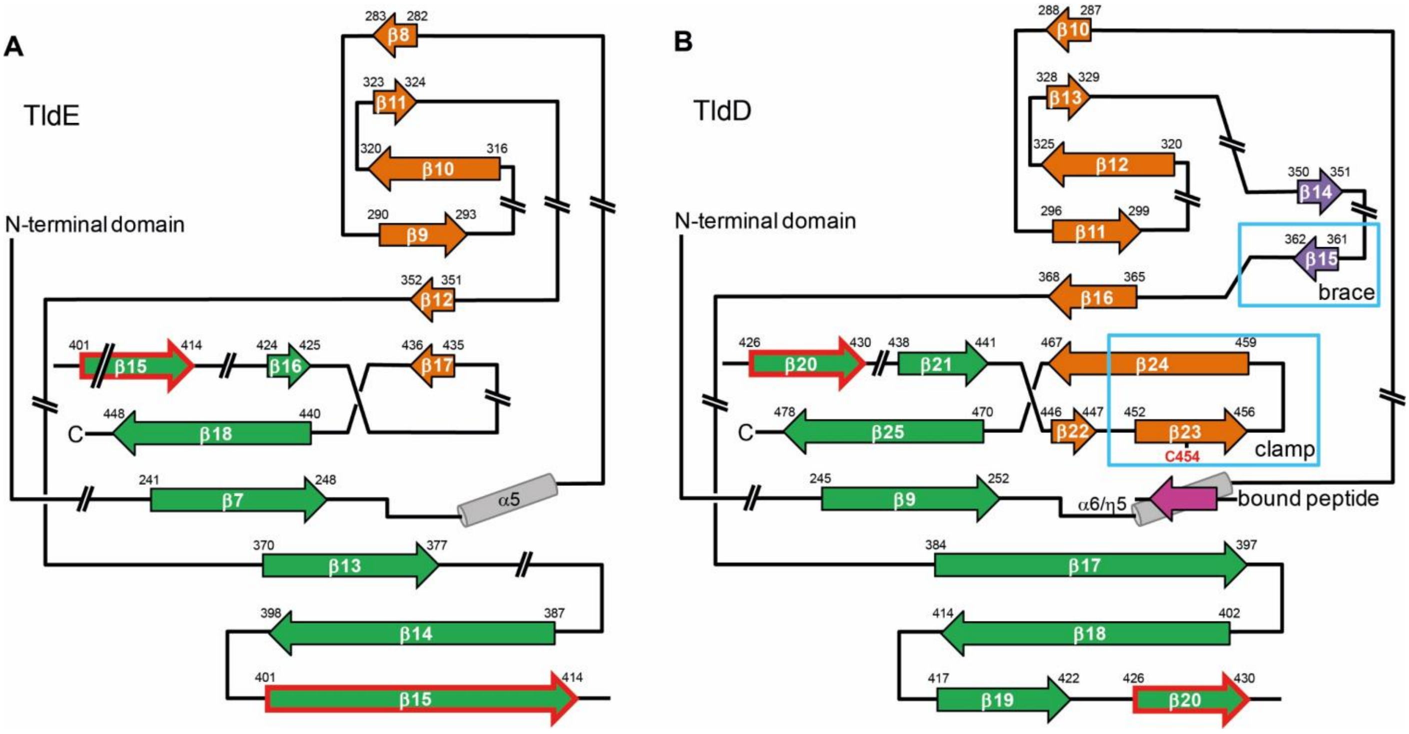


**Figure S5. Topology diagrams for C-terminal domains of TldE and TldD. Related to Figures 3, 5 and 7** (**A**) Arrangement and connectivity of β-strands in TldE showing only the central α-helix (α5). The strands are arranged into a mainly anti-parallel β-sheet (orange arrows) and an anti-parallel β-barrel (green arrows; β15 is shown twice, outlined in red, to indicate the continuity of the β-barrel hydrogen bonding network). (**B**) Equivalent diagram for TldD. Note the additional β-hairpin that forms as a result of the brace insertion (purple arrows), and the clamp insertion that forms one side of the active site cleft (the position of the zinc ligand Cys454 is indicated). When there is a peptide bound in the active site (magenta arrow), this effectively unites the β-sheet (orange arrows) and the anti-parallel β-barrel (green arrows) though anti-parallel hydrogen bonding interactions (see Figure 5**E**). For both panels, residue numbers defining the start and end of each β-strand are shown, and double slashes indicate where connections have been foreshortened.

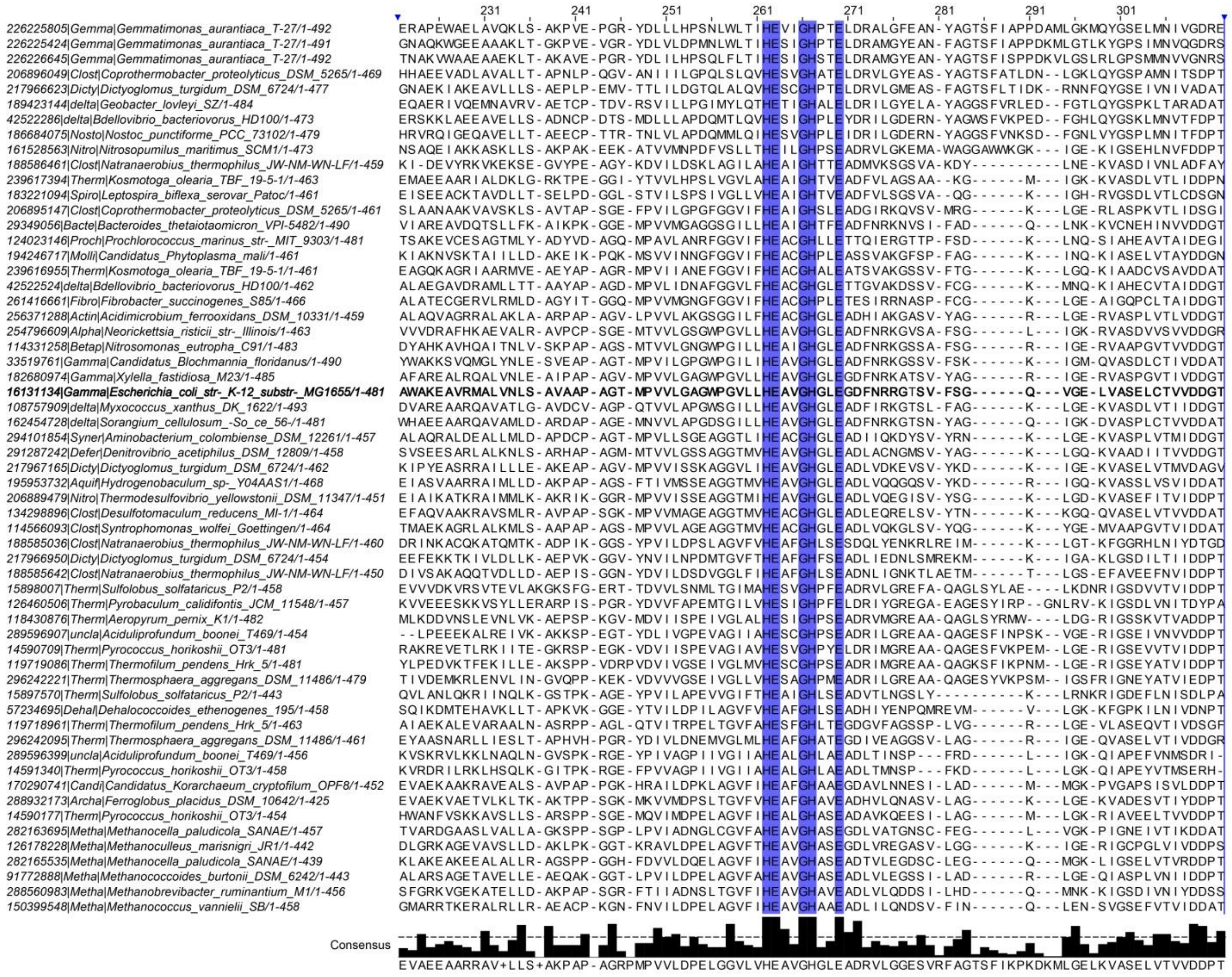

**Figure S6. Multiple sequence alignment of a non-redundant set of TldD proteins**. **Related to Figures 3 and 4.** Sequences of TldD proteins from GenBank were aligned with ClustalOmega (http://www.clustal.org/omega/) (Sievers et al., 2011) and visualized using JalView (http://www.jalview.org) (Waterhouse et al., 2009)

*E. coli* MG1655 numbering is used for convenience. Only part of the alignment is shown, representing the conserved HExxGHxxE motif found in TldD (100% conservation), highlighted in blue.

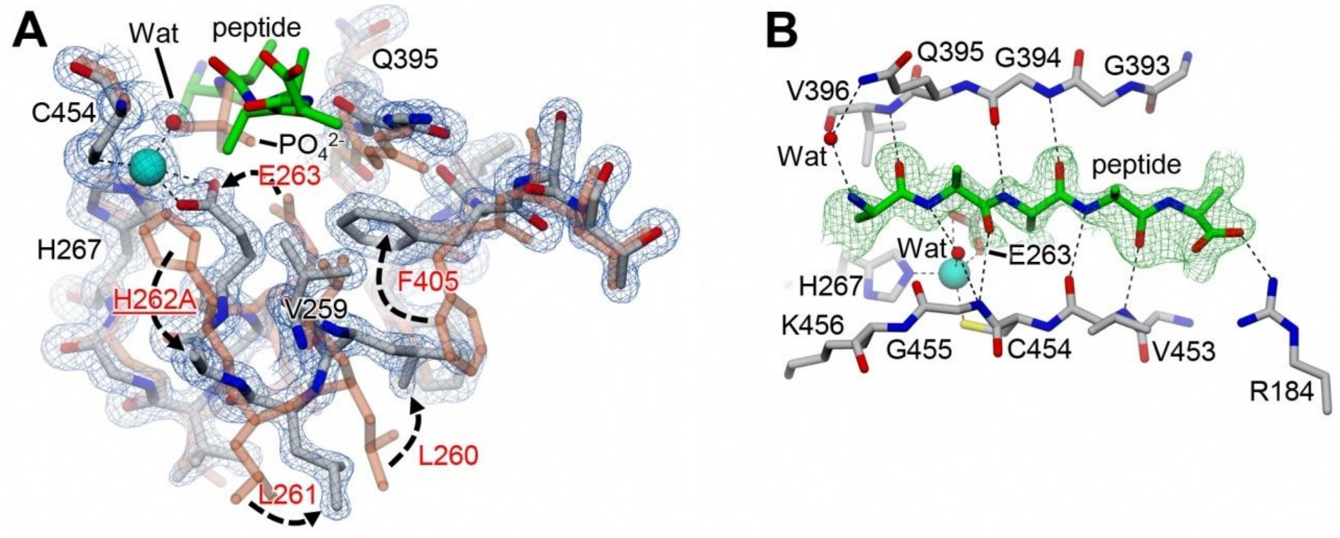


**Figure S7. Detail of the TldD H262A mutant. Related to Figures 3 and 4.** (**A**) The metal binding site and its immediate environment is reconfigured as a result of the H262A substitution (H262A-pent dataset, Tables 1 and 2). The view shown is looking along the active site cleft from the direction of the entrance channel. The mutant protein structure (atom colouring; grey carbons; zinc – cyan sphere) with a poly-Ala pentapeptide (green carbons; see part **B**), is compared to that of the wild-type structure with phosphate bound to the catalytic zinc (pale salmon atoms; semi-transparent). Final electron density (2*mFobs-dFcalc*; 1.42 Å resolution; contoured at ~1.0σ) is shown for all displayed atoms of the mutant structure with the exception of the ligand. Key residues are labelled, with those showing the largest shifts in red, where the corresponding changes are indicated by the black dashed arrows; the direction of the arrow shows the movement on transforming from wild-type to mutant. The loss of the imidazole moiety at residue 262 (underlined) is compensated for by the movement of the Glu263 carboxylate group to become a new zinc ligand. This causes a rotation of the segment including residue 263 and the preceding three residues. The movement of Leu260 displaces the side-chain of Phe405 in β18, to occupy the space vacated by Glu263. The upward movement of Phe405 also displaces β17 slightly (which includes Gln395). (**B**) Weak omit electron density (*mFobs-dFcalc*; 1.42 Å resolution; contoured at ~2.5σ) is present in the active site cleft of the TldD H262A mutant, which has been modelled and refined as a poly-Ala pentapeptide with an occupancy of 0.5; the density was not sufficiently well defined to allow identification of any of the residues of the ligand. The view shown is looking down on the active site cleft and corresponds to that presented for the TldD E263A mutant in Figure 5**E**. The pentapeptide shown here superposes well on the hexapeptide observed in the equivalent position in the latter mutant. In conclusion, this structure reveals how the TldD H262A mutant is able to retain wild-type metal content by recruiting Glu263 to compensate for the loss of the H262 side-chain. However, it is inactive because Glu263 is no longer available to perform its key role in the proteolytic mechanism.

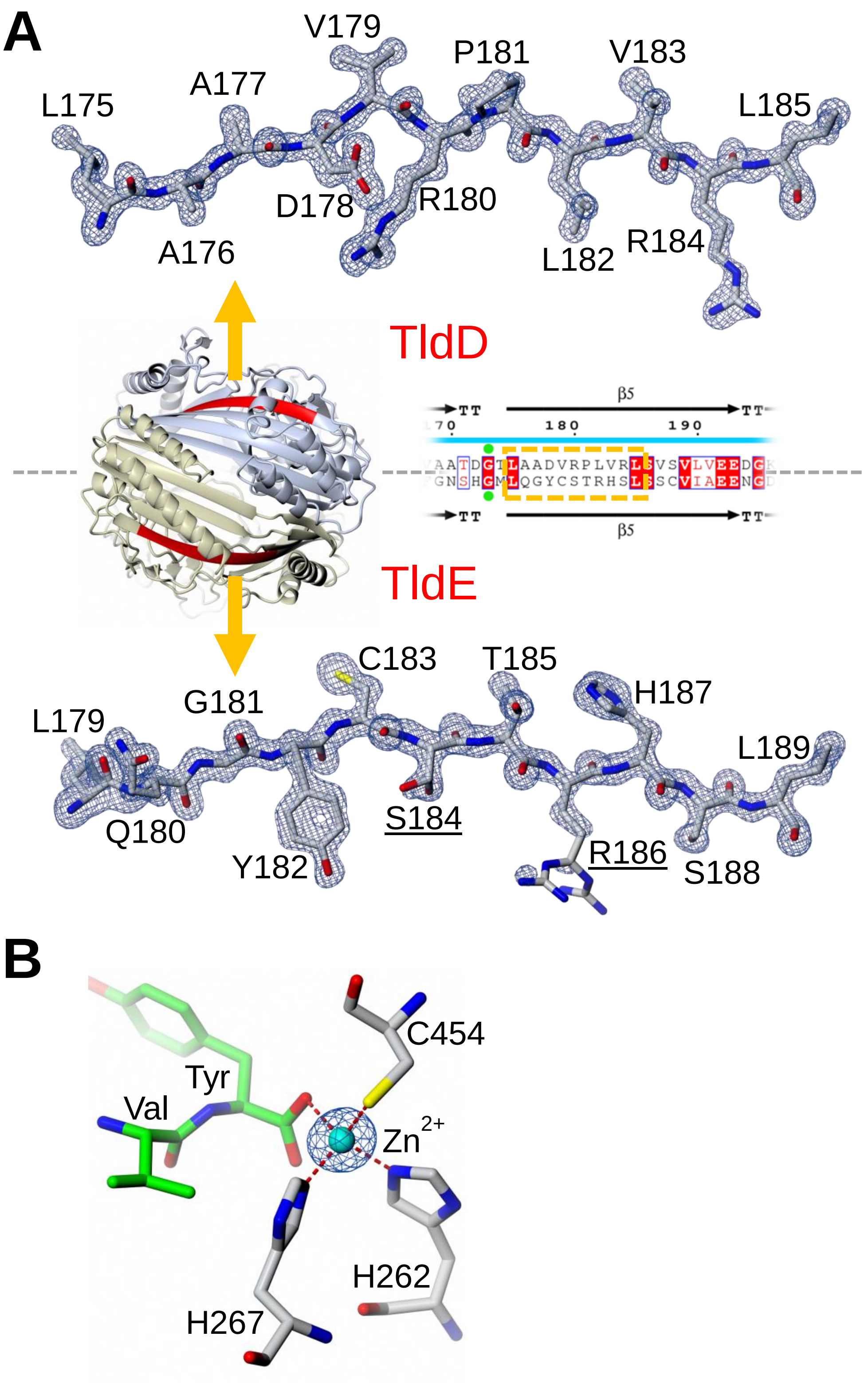


**Figure S8. Validation of components of TldDE structure. Related to Figures 3 and 4.** (**A**) To verify that the structures we present contain TldDE heterodimers, we located a structurally equivalent section from the two monomers (coloured red in the cartoon overview), where the TldD and TldE sequences were very different. This was an eleven-residue peptide within β8 consisting of two conserved Leu residues framing nine non-conserved residues [highlighted by the dashed orange box in the inset sequence alignment (section of Figure S4)]. These eleven residue sequences were deleted from the final coordinates

of the TldDE complex with cleaved angiotensin (WT-DRVY dataset) before calculating an omit difference density map using the procedure described in the Methods. Shown at the top and bottom in stick representation are the final coordinates of the peptides that were deleted from TldD and TldE, respectively, superposed on the resultant 1.25 Å resolution omit difference electron density (contoured at ~4σ). The latter is of sufficient quality to confirm the identities of the two subunits. The density is weaker for Ser184 and Arg186 in TldE (underlined), which were both modelled in two alternate conformations. (**B**) At the wavelength of the X-rays used to collect the data for this complex (λ = 0.9795), zinc is expected to have a measurable anomalous signal (~2.5 anomalous electrons). Thus, an anomalous difference Fourier map shows a clear peak for the tetrahedrally bound zinc (calculated at 3.0 Å resolution and contoured at ~15σ). The TldD liganding residues are shown with grey carbons and part of the bound angiotensin peptide is shown with green carbons.
